# Two Novel Quassinoid Glycosides with Antiviral Activity from the Samara of *Ailanthus altissima*

**DOI:** 10.3390/molecules25235679

**Published:** 2020-12-02

**Authors:** Qing-Wei Tan, Jian-Cheng Ni, Jian-Ting Shi, Jian-Xuan Zhu, Qi-Jian Chen

**Affiliations:** 1 Key Laboratory of Biopesticide and Chemical Biology, Ministry of Education, Fujian Agriculture and Forestry University, Fuzhou 350002, China; shijianting00@163.com (J.-T.S.); 17793649297@163.com (J.-X.Z.); 2 The Engineering Technology Research Center of Characteristic Medicinal Plants of Fujian, Ningde Normal University, Ningde 352100, China; njc2130215001@163.com

**Keywords:** *Ailanthus altissima* (Mill.) Swingle, Simaroubaceae, quassinoid, lignanoid, tobacco mosaic virus

## Abstract

Phytochemistry investigations on *Ailanthus altissima* (Mill.) Swingle, a Simaroubaceae plant that is recognized as a traditional herbal medicine, have afforded various natural products, among which C_20_ quassinoid is the most attractive for their significant and diverse pharmacological and biological activities. Our continuous study has led to the isolation of two novel quassinoid glycosides, named chuglycosides J and K, together with fourteen known lignans from the samara of *A. altissima*. The new structures were elucidated based on comprehensive spectra data analysis. All of the compounds were evaluated for their anti-tobacco mosaic virus activity, among which chuglycosides J and K exhibited inhibitory effects against the virus multiplication with half maximal inhibitory concentration (IC_50_) values of 56.21 ± 1.86 and 137.74 ± 3.57 μM, respectively.

## 1. Introduction

Different parts of *Ailanthus altissima* (Mill.) Swingle, a Simaroubaceae plant native to China and widely distributed in North America and European countries [1,2], have been extensively used in traditional Chinese medicine for the treatments of ascariasis, bleeding, spermatorrhea, diarrhea and gastrointestinal diseases [3]. The extract of the plant has been reported to possess a broad range of bioactivities such as antitumor, antimalarial, antiviral, antiparasitic, herbicidal and insecticidal properties [4,5,6]. Phytochemical investigations have reported the characterization of quassinoids, β-carboline alkaloids, triterpenoids, coumarins, phenylpropionamides, piperidine, and phenolic derivatives [7,8,9,10,11,12], etc. We have initiated a phytochemistry study on the samara of *A. altissima*, and previously we have reported the identification of 58 natural products of diverse structures such as quassinoids, coumarins, flavonoids, phenylpropanoids, phenylpropionamides, piperidine and phenolic derivatives, among which were a series of novel compounds including nine quassinoid glycosides, two phenylpropionamides, one piperidine, one terpenylated coumarin and two phenolic derivatives [5,7,13]. In this paper, we report the purification and identification of another two novel quassinoid glycosides, named chuglycosides J (**1**) and K (**2**), which were isolated from the *A. altissima* samara during our ongoing studies, together with fourteen known lignan and neolignans including tetrahydro-2-(4-hydroxy-3-methoxyphenyl)-4-[(4-hydroxyphenyl) methyl]-3-furanmethanol (**3**) [14], (+)-lariciresinol (**4**) [15], (+)-(1*R*,2*S*,5*R*,6*S*)-2,6-di(4′-hydroxyphenyl)-3,7-dioxabicyclo [3.3.0]octane (**5**) [16], (+)-pinoresinol (**6**) [17], (+)-isolariciresinol (**7**) [18], (+)-isolariciresinol 3α-*O*-β-glucopyranoside (**8**) [19], burselignan (**9**) [18], densispicoside (**10**) [20], secoisolariciresinol (**11**) [15], dehydroconiferyl alcohol (**12**) [21], curcasinlignan B (**13**) [22], erythro-guaiacylglycerol-β-*O*-4′-coniferyl ether (**14**) [23], 7*R*,8*R*-threo-4,7,9,9′-tetrahydroxy-3,3′-dimethoxy-8-*O*-4′-neolignan (**15**) [24], threo-2,3-bis-(4-hydroxy-3-methoxyphenyl)-3-methoxypropanol (**16**) [25]. The structures of two novel quassinoid glycosides were elucidated based on the analysis of their chemical properties and spectroscopic data, and all of the compounds obtained were evaluated for their antiviral effect against tobacco mosaic virus in vitro.

## 2. Result

### 2.1. Extraction, Isolation and Sructure Elucidation

**Figure 1:**
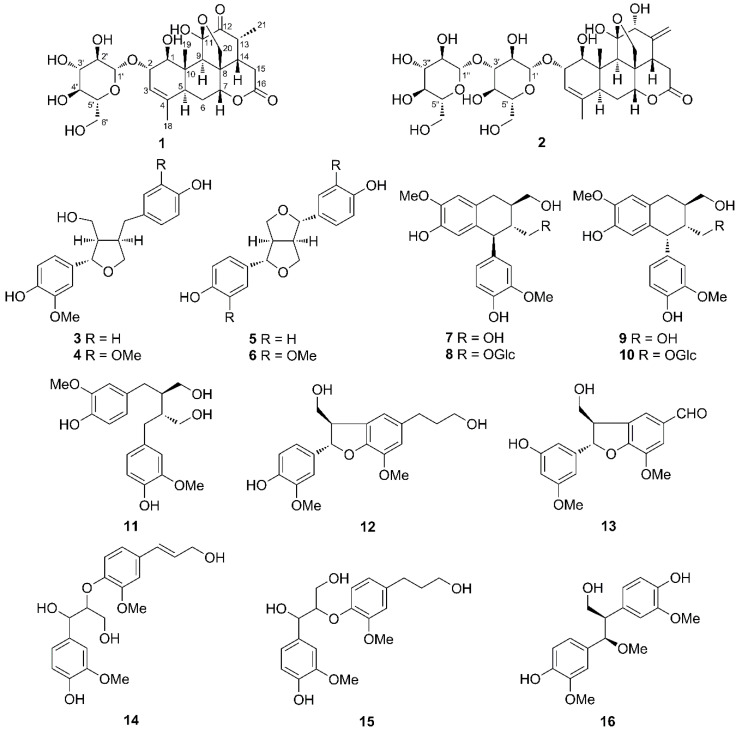
Chemical structures of compounds **1**–**16**.

The air-dried and milled samara of *A. altissima* was extracted with methanol to afford a crude extract, which was resuspended in water and fractioned by liquid-liquid partition. From the trichloromethane partition, a total of ten lignans were isolated, while compounds **1** and **2**, together with **4**, **6**, **9** and **16,** were purified from the *n*-butyl alcohol partition (Figure 1).

**Figure 2:**
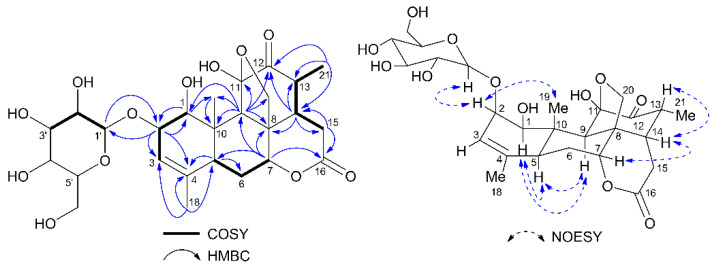
Selected HMBC (arrows), ^1^H-^1^H COSY (bold lines) and key NOESY (dashed arrows) correlations of compound **1**.

Compound **1** was isolated as a colorless crystal, and its molecular formula was established as C_26_H_36_O_12_ by high resolution ionization mass spectroscopy (HRESIMS) (*m*/*z* 564.2175 [M + Na + H]^+^, calcd for C_26_H_37_O_12_Na 564.2177) (Figure S1). Its IR spectrum (Figure S2) displayed absorption bands indicating the presence of hydroxyl (3427 cm^−1^), δ-lactone (1731 cm^−1^) and double bond (1640 cm^−1^). The ^1^H-NMR (Figure S3) spectra of **1** displayed signals for a characteristic glycopranosyl moiety [δ_H_ 4.31 (1H, d, *J* = 7.7 Hz, H-1′), 3.70–3.62 (1H, overlap, H_a_-6′), 3.43 (1H, dt, *J* = 11.7, 5.9 Hz, H_b_-6′), 3.21–3.08 (2H, overlap, H-3′ and H-5′), 3.02 (1H, td, *J* = 9.3, 4.1 Hz, H-4′), 2.95 (1H, m, H-2′)], as well as signals for an olefinic [δ_H_ 5.65 (1H, m)], three oxygenated methine [δ_H_ 4.58 (1H, t, *J* = 2.8 Hz, H-7), 3.95–3.87 (1H, overlap, H-2), 3.69 (1H, d, *J* = 8.1 Hz, H-1), one oxygenated methylene [δ_H_ 4.16 and 3.90 (each 1H, d, *J* = 8.9 Hz, H_2_-20)], four methine [δ_H_ 2.24 (1H, br d, *J* = 12.5 Hz, H-5), 1.99 (1H, s, H-9), 3.21–3.08 (1H, overlap, H-13), 2.59 (1H, ddd, *J* = 12.4, 8.5, 7.0 Hz, H-14)], two methylene [δ_H_ 2.36–2.30 (2H, overlap, H-15), 1.94 (1H, td, *J* = 14.8, 2.6 Hz, H_a_-6) and 1.86 (1H, dt, *J* = 14.8, 2.9 Hz, H_b_-6)], and four methyl groups [δ_H_ 1.61 (3H, s, H_3_-18), 1.17 (3H, s, H_3_-19), 0.86 (3H, d, *J* = 6.7 Hz, H_3_-21)]. A combined analysis of the ^13^C- (Figure S4) and distortionless enhancement by polarization transfer (DEPT) (Figure S5) NMR spectra revealed that compound **1** has 26 carbons including two carbonyl (δ_C_ 207.7 and 169.1), two olefinic carbons (δ_C_ 134.6 and 124.2), one hemiketal carbon (δ_C_ 106.9), six saccharide-type carbons (δ_C_ 105.2, 76.7, 76.3, 74.2, 70.0 and 61.1), as well as three methyl, three methylene, seven methine, and two quaternary carbons. The above ^1^H and ^13^C-NMR data were similar to those of the quassinoid glycosides isolated from the same plant materials, as reported previously in our paper [13]. Specifically, a keto group was attached at C-12 position, as indicated by the observed HMBC correlations (Figure 2) between H-21 [δ_H_ 0.86 (3H, d, *J* = 6.7 Hz)] and C-12 (δ_C_ 207.7), H-9 [δ_H_ 1.99 (1H, s)] and C-12, H-13 [δ_H_ 3.21–3.08 (1H, overlap)] and C-12, and between H-14 [δ_H_ 2.59 (1H, ddd, *J* = 12.4, 8.5, 7.0 Hz)] and C-12. Acid hydrolysis of **1** afforded d-glucose, and the HMBC correlations between the anomeric proton H-1′ [δ_H_ 4.31 (1H, d, *J* = 7.7 Hz)] and C-2 (δ_C_ 82.9), and between H-2 [δ_H_ 3.95–3.87 (1H, overlap)] and C-1′ (δ_C_ 105.2) confirmed that the glucopyranosyl unit was attached at the C-2 position, and it must be a β-anomer as suggested by the coupling constant of the anomeric proton. The NOESY cross-peaks (Figure 2) of H-1′/H-2, H-2/H_3_-19, H-7/H-14, and H-14/H-21 indicated that H-1′, H-2, H-7, H-14, H-21 and Me-19 were cofacial and assigned as β-orientations. While the NOESY correlations of H-1/H-5, H-5/H-9, and H-1/H-9 showed that these protons were α-oriented. Therefore, the structure of **1** was characterized as 11β,20-epoxy-1β,11α-dihidroxypicrasa-3-ene-12,16-dione-2-*O*-β-d-glucopyranoside, and named as chuglycoside J.

**Figure 3:**
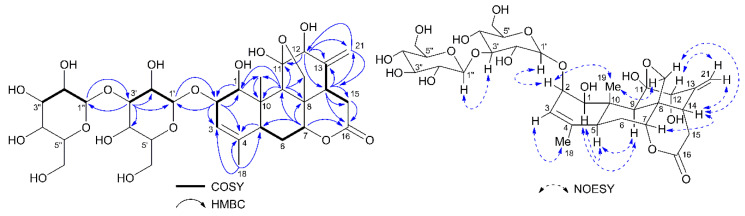
Selected HMBC (arrows), ^1^H-^1^H COSY (bold lines) and key NOESY (dashed arrows) correlations of compound **2**.

Compound **2** was purified as a colorless crystal with a molecular formula of C_32_H_46_O_17_ as shown by a sodiated molecular ion peak at *m*/*z* 725.2656 (calcd for C_32_H_46_O_17_Na 725.2627) observed with HRESIMS (Figure S10). The IR spectrum (Figure S11) displayed absorption bands indicating the presence of hydroxyl (3398 cm^−1^), δ-lactone (1718 cm^−1^) and double bond (1648 cm^−1^). Its ^13^C-NMR and DEPT spectra (Figures S13 and S14) showed 32 carbon resonances, including two methyl, six methylene, eighteen methine, and six quaternary carbons. Compound **2** was also a quassinoid glycoside as deduced from a comparison of its ^1^H and ^13^C-NMR data with those of compound **1**, as well as the analysis of its DEPT, COSY, HSQC, HMBC, and NOESY spectra (Figures S13–S18). Signals of a terminal double bond [δ_H_ 5.22 (br s, 2H, H-21); δ_C_ 120.3 (C-21)] were observed in its ^1^H-NMR and ^13^C-NMR spectra (Figures S12 and S13), and the HMBC correlations (Figure 3) observed between H-12 [δ_H_ 3.89 (1H, s)] and C-21, between H-14 [δ_H_ 2.80 (dd, *J* = 13.5, 5.4 Hz, 1H) and C-21, between H-21 and C-12 (δ_C_ 81.0), as well as HMBC correlations between H-21 and C-14 (δ_C_ 48.0). Two anomeric protons appearing at δ_H_ 4.60 (d, *J* = 7.8 Hz, 1H, H-1′) and 4.56 (d, *J* = 7.9 Hz, 1H, 1′′) in the ^1^H-NMR spectra indicated the presence of two glucopyranosyl units, both of which must be β-anomer as suggested by their coupling constant. Acid hydrolysis of **2** afforded only d-glucose, which was identified by thin-layer chromatography (TLC) comparisons with sugar standards, and the HMBC correlations between the anomeric proton H-1′ and C-2 (δ_C_ 84.1), between H-2 [δ_H_ 4.19 (1H, m)] and C-1′ (δ_C_ 105.0), and between the anomeric proton H-1′′ and C-3′ (δ_C_ 88.1), between H-3′ [δ_H_ 3.58 (1H, t, *J* = 8.9 Hz)] and C-1′′ (δ_C_ 105.2) confirmed that two glucopyranosyl units were connected via a (1→3) linkage, and the saccharide moiety was attached at the C-2 position. The NOESY cross-peaks (Figure 3) of H-1′/H-2, H-2/H_3_-19, H-7/H-14, and H-14/H-21 indicated that H-1′, H-2, H-7, H-12, H-14, and Me-19 were cofacial and assigned as β-orientations. While the NOESY correlations (Figure 3) of H-1/H-5, H-5/H-9, and H-1/H-9 showed that these protons were α-oriented. Compound **2** was established as 11β,20-epoxy-1β,11α,12α-trihidroxypicrasa-3,13(21)-diene-16-one-2-*O*-β-d-glucopyranosyl-(1→3)-β-d-glucopyranoside, and named as chuglycoside K.

### 2.2. Antiviral Activities against the Replication of Tobacco Mosaic Virus

The isolated compounds were tested for their inhibitory activities against the replication of tobacco mosaic virus using the leaf-disc method. The lignans (**3**–**16**) obtained showed only weak or no inhibitory effect at a concentration of 0.5 mM (Table 1). Both of the quassinoid glycosides obtained, chuglycoside J (**1**) and K (**2**), exhibited antiviral activities with IC_50_ values determined as 56.21 ± 1.86 and 137.74 ± 3.57 μM, while the commercial antiviral agents, ningnanmycin and ribavirin, possessed an IC_50_ of 183.31 ± 4.26 and 255.19 ± 4.57 μM, respectively.

## 3. Discussion

Quassinoids, one kind of degraded triterpenoid derivative with multiple bioactivities such as anticancer, antimalarial, antimicrobial, antidiabetic, antiviral, and anti-inflammatory effects, are widely distributed in the family Simaroubaceae and the secondary metabolites characteristic of this family [26,27,28]. Quassinoids can generally be classified into five groups according to the basic features, including C_18_, C_19_, C_20_, C_22_ and C_25_ types. By the year 2004, more than 200 natural quassinoids obtained from 34 species in 14 genera were reported, and the structural characteristics of 190 quassinoids were reported between the year 2004 and 2018 [27,28]. Thus far, more than 50 quassinoids have been isolated from *A. altissima*, most of which belong to the C_20_ class bearing a δ-lactone moiety [13,26]. Pharmacological and clinical investigations have revealed that C_20_ quassinoids from *Ailanthus* genus plant are very promising for their medical use, such as antitumor, antimalarial, antiviral, antiparasitic properties etc. [4,5,6]. We have previously reported the identification of eighteen C_20_ quassinoids including nine new quassinoid glycosides, named chuglycosides A–I, from the samara of *A. altissima*. The generated name chuglycoside arises from ‘chu’, the Chinese phonetic alphabets of one of the variant names of *A. altissima*, which is commonly used in the classics of traditional Chinese medicine. The quassinoids previously obtained from the samara of *A. altissima* can generally be classified as the C-6 or C-15 substituted derivatives of chaparrione and ailanthone, as well as their monoglycosides [13]. Ailanthone is the best known bioactive secondary metabolite isolated from *A. altissima*, which displays multiple pharmacological properties, in particular significant antitumor effects against a variety of cancer cell lines in vitro [29,30]. Two more C_20_ quassinoid glycosides were reported based on our current findings, among which chuglycoside J wears a keto group in C-12, which, in the case of quassinoids from *A. altissima,* usually appears as a hydroxy substituted group, while chuglycoside K was the only one diglycoside we obtained from the extract of the samara of *A. altissima*. These findings suggest that phytochemical investigations to reveal the structural diversity of quassinoids synthesized by *A. altissima* are still worth undertaking.

In the field of modern agriculture, plant diseases caused by viruses are one of the major causes of biological disasters in agriculture and horticulture, which result in dramatic losses every year all over the world. The research and development of efficient antiviral agents with characteristics of low pesticide resistance, eco-friendliness and a novel mechanism is urgently and continuously needed [31,32]. Tobacco mosaic virus, the type member of the Tobamovirus group, is a positive-strand RNA virus that infects more than 400 plant species belonging to 36 families, such as tobacco, tomato, potato, and cucumber [33,34,35,36,37]. The tobacco mosaic virus/tobacco system is employed as a useful model in studies designed to clarify antiviral properties and the mechanism of action of novel antiviral agents. Our continuous efforts regarding the discovery of novel anti-phytopathogen viruses from natural products and studies of the mechanisms of antiviral action have proven that quassinoids from *A. altissima* could inhibit the coat protein expression and systemic spread of tobacco mosaic virus in tobacco [13] and this evidence has proven that quassinoids from *A. altissima* could be considered as lead structures for antiviral agent design and development.

## 4. Materials and Methods

### 4.1. Chemical Studies

All the solvents used for column chromatography were of analytical-reagent grade and purchased from Sinopharm Chemical Reagent Co., Ltd., Shanghai, China. IR spectra were acquired with a Thermo Scientific Nicolet iS50 FT-IR spectrometer (Thermo Scientific, Waltham, MA, USA). ^1^H- and ^13^C-NMR, DEPT 90, DEPT 135, HSQC, ^1^H-^1^H COSY, HMBC and NOESY spectra were recorded on a Bruker AVANCE III 500 spectrometer (Bruker BioSpin, Fällanden, Switzerland). HRESIMS were collected on an Agilent 6520 Q-TOF mass spectrometer (Agilent Technologies, Santa Clara, CA, USA). Column chromatography was conducted using silica gel (200–300 or 300–400 mesh), Sephadex LH-20 (Pharmacia Fine Chemical Co., Ltd., Uppsala, Sweden), MCI-gel CHP-20P (75–150 μm, Mitsubishi Chemical Co., Tokyo, Japan) and Lichroprep RP-18 gel (40–63 μm, Merck, Darmstade, Germany), while analytical thin-layer chromatography (TLC) was performed on glass-backed plates precoated with 0.25 mm layers of Silica Gel H (Qingdao Oceanic Chemical Co., Qingdao, China) and visualized by heating silica gel plates sprayed with 5% H_2_SO_4_ in ethanol.

### 4.2. Extraction and Isolation

The air-dried samara of *Ailanthus altissima* was collected in October 2013 at Muyang, Jiangsu, China. The voucher specimen was deposited at the Key Laboratory of Biopesticide and Chemical Biology, Ministry of Education, Fujian Agriculture and Forestry University, under the accession number MF131001.

The plant material was extracted and partitioned as described previously in our paper [13]. In brief, the milled air-dried samara of *A. altissima* (sample MF131001, 7500 g) was extracted three times with a total of 25 L methanol at room temperature. The dried extract was then resuspended in water and successively partitioned with *n*-hexane, trichloromethane, and *n*-butyl alcohol. 

The chloroform extract (30 g) was fractioned by silica gel column chromatography and eluted with gradient mixtures of 0 to 100% methanol in chloroform to give eleven fractions (fraction B1–B11). Fraction B5 (1.3 g) was subjected to Sephadex LH-20 column chromatography (always conducted using a mixture solvent of 50% trichloromethane in methanol as mobile phase) to give five fractions (fractions B5a–B5e). Fraction B5c was further purified by column chromatography using Sephadex LH-20 to give compound **12** (15.0 mg). Fraction B5d was separated using RP-18 gel column chromatography and eluted with a mixture of 70% methanol in water, and further purified by chromatographing using a silica gel column and eluted with CHCl_3_–MeOH (*v/v* 98:2) to yield **13** (7.0 mg). Fraction B6 (972 mg) was separated by Sephadex LH-20 column chromatography to give fractions B6a–B6e. Fraction B6c was chromatographed over RP-18 gel column chromatography and eluted with 60% methanol in water to give **11** (5.0 mg). Fraction B7 (1.4 g) was chromatographed using a MCI gel column and eluted with a gradient of 50–100% methanol in water to afford fractions B7a–B7j. Fractions B7d, B7e and B7f were, respectively, chromatographed over a silica gel column and eluted with CHCl_3_–MeOH (*v/v* 98:2) to yield **7** (19.0 mg), **14** (26.9 mg) and **15** (76.0 mg). Fraction B8 (990 mg) was separated by RP-18 gel column chromatography and eluted with a gradient of 50–100% methanol in water to provide fractions B8a–B7i. Fractions B8e was chromatographed over a silica gel column and eluted with CHCl_3_–MeOH (*v/v* 98:2) to yield **10** (59.0 mg). Fraction B9 (645 mg) was subjected to MCI gel column chromatography and eluted with a gradient of 40–100% methanol in water to afford fractions B9a–B9e. Fraction B9e was chromatographed over a silica gel column and eluted with CHCl_3_–MeOH (*v/v* 97:3) to yield **3** (367.7 mg). Fraction B10 (1.9 g) was separated using RP-18 gel column chromatography and eluted with a gradient of 40% to 100% water in MeOH to afford fractions B10a–B10g. Fraction B10f was then chromatographed over a silica gel column and eluted with CHCl_3_–MeOH (*v/v* 98:2) to yield **5** (6.0 mg) and **8** (28.0 mg). 

The *n*-butyl alcohol partition (90 g) was fractioned by silica gel column chromatography and eluted with mixtures of 0 to 100% methanol in chloroform to give fourteen fractions (fraction C1–C14). Fraction C2 was subjected to MCI gel column chromatography and eluted with a gradient of 15–100% water in MeOH to afford fractions C10a–C10e. Fraction C10e was chromatographed over a silica gel column and eluted with CHCl_3_–MeOH (*v/v* 97:3) to yield **16** (5.5 mg). Fraction C3 (3.5 g) was separated by MCI gel column chromatography and eluted with a gradient of 15–100% methanol in water to afford fractions C3a–C3p. Fraction C3i was chromatographed over a silica gel column using a mixture of CHCl_3_–MeOH (96:4) as eluent to yield **9** (22.0 mg). Fraction C5 (2.2 g) was chromatographed using a MCI gel column and eluted with a gradient of 15–100% methanol in water to afford fractions C5a–C5e. Fraction C5c was purified using RP-18 gel column chromatography with a solvent of 30% methanol in water as eluent to give **2** (5.0 mg). Fraction C8 (10.1 g) was subjected to MCI gel column chromatography and eluted with a gradient of 5–100% water in MeOH to afford fractions C8a–C8k. Fraction C8c was subjected to RP-18 gel column chromatography and eluted with 30% MeOH in water in to give **4** (70.9 mg). Fraction C10 (10.1 g) was subjected to MCI gel column chromatography and eluted with a gradient of 5–100% methanol in water to afford fractions C10a–C10h. Fraction C10e was further purified using RP-18 gel column chromatography and eluted with 30% methanol in water in to afford **6** (11.5 mg). Fraction C11 (10.3 g) was chromatographed using a MCI gel column and eluted with a gradient of 5–100% methanol in water to afford fractions C11a–C11h. Fraction C11e was then separated using RP-18 gel column chromatography and eluted with a mixture of 45% MeOH in water and finally chromatographed over a silica gel column and eluted with CHCl_3_–MeOH–H_2_O (*v/v/v* 80:20:2) to yield **1** (7.1 mg).

#### 4.2.1. Chuglycoside J (**1**)

Colorless crystal; melting point (mp) 199–201 °C; ${\left[\alpha \right]}_{D}^{20}$ +6.43 (*c* 0.1, MeOH); IR (KBr) *υ*_max_: 3427, 2975, 2892, 1731, 1640, 1503, 1436, 1387, 1250, 1154, 1134, 1076, 1051, 980, 905 cm^−1^; positive-ion HRESIMS *m*/*z* 564.2175 [M + Na + H]^+^ (calcd for C_26_H_37_O_12_Na 564.2177); ^13^C- and ^1^H-NMR data see Table 2.

#### 4.2.2. Chuglycoside k (**2**)

Colorless crystal; mp 228–229 °C; ${\left[\alpha \right]}_{D}^{20}$ +30.0 (*c* 0.1, MeOH); IR (KBr) *υ*_max_: 3398, 2884, 1718, 1648, 1440, 1382, 1324, 1254, 1154, 1038, 1013, 984, 905 cm^−1^; HR-ESI-MS *m*/*z* 725.2656 [M + Na]^+^ (calcd for C_32_H_46_O_17_Na 725.2627); ^13^C- and ^1^H-NMR data see Table 2.

### 4.3. Acid Hydrolysis of Compounds ***1*** and ***2***

Compound **1** or **2** (each 2 mg) was hydrolyzed at 95 °C for 2 h in 2 mL of 1 M HCl (dioxane–H_2_O, *v/v* 1:1), respectively. After being evaporated to dryness, the reaction mixtures were diluted in water and extracted with 2 mL ethyl ether three times. The aqueous layer was neutralized with NaHCO_3_ and evaporated under vacuum to furnish a neutral residue for thin-layer chromatography (TLC) analysis, which indicated the presence of only d-glucose (*Rf* 0.40; eluted with MeCOEt–isoPrOH–Me_2_CO–H_2_O, *v/v* 20:10:7:6).

### 4.4. Antiviral Assay

The isolated quassinoids and lignans were dissolved in dimethyl sulfoxide (DMSO) and diluted to the required concentration before the test. Two commercial agents, ningnanmycin and ribavirin, were used as positive control agents, while a solution of 0.01 M phosphate-buffered saline (PBS) containing 1% DMSO was used as negative control. Purified tobacco mosaic virus (TMV) U1 strain was obtained from the Institute of Plant Virology, Fujian Agriculture and Forestry University. *Nicotiana tabacum* cv. K_326_, which were cultivated to 5–6 leaf stage in an insect-free greenhouse, were used for the anti-tobacco mosaic virus (TMV) assay. 

The antiviral assay was conducted using the leaf-disc method as previously described in our paper [5,7,13,38,39]. In brief, the growing leaves of tobacco were mechanically inoculated and infected with the target virus. Six hours later, leaf discs of 1 cm diameter were punched and floated on the test solutions, while leaf discs from the healthy leaves were used as mock. Six replicates were carried out for each sample. The test solutions with leaf discs were kept in a Petri dish and incubated for 48 h at 25 °C in a culture chamber, and then the leaf discs were grounded with the addition of 0.01 M pH 9.6 carbonate coating buffer (500 μL for each leaf disc) and centrifuged. The supernatant of each sample (200 μL) was transferred to a 96-well plate, which was then used to perform a standard indirect enzyme-linked immunosorbent assay as described in the literature [34,35]. The virus concentration was calculated according to a standard curve constructed based on the optical density at 405 nm (OD_405_) values of a series of the diluted solutions of purified TMV.

## 5. Conclusions

Continuous phytochemical investigations on the samara of *A. altissima* led to the identification of two novel quassinoid glycosides, chuglycosides J and K, together with fourteen known lignans including tetrahydro-2-(4-hydroxy-3-methoxyphenyl)-4-[(4-hydroxyphenyl) methyl]-3-furanmethanol, (+)-lariciresinol, (+)-(1*R*,2*S*,5*R*,6*S*)-2,6-di(4′-hydroxyphenyl)-3,7-dioxabicyclo[3.3.0]octane, (+)-pinoresinol, (+)-isolariciresinol, (+)-isolariciresinol 3α-*O*-β-glucopyranoside, burselignan, densispicoside, secoisolariciresinol, dehydroconiferyl alcohol, curcasinlignan B, erythro-guaiacylglycerol-β-*O*-4′-coniferyl ether, 7*R*,8*R*-threo-4,7,9,9′-tetrahydroxy-3,3′-dimethoxy-8-*O*-4′-neolignan, threo-2,3-bis-(4-hydroxy-3-methoxyphenyl)-3-methoxypropanol, among which two quassinoids glycosides showed noticeable inhibitory effects against the replication of tobacco mosaic virus.

## Figures and Tables

**Table 1:** Inhibitory activity of 3–16 against the replication of tobacco mosaic virus.

Compounds ^a^	Inhibitory Rate (%, Mean Value ± SD)
**3**	–
**4**	12.8 ± 4.7
**5**	37.6 ± 4.2
**6**	22.5 ± 4.4
**7**	–
**8**	–
**9**	66.5 ± 2.8
**10**	58.2 ± 5.2
**11**	13.0 ± 2.8
**12**	19.3 ± 4.0
**13**	13.8 ± 1.7
**14**	–
**15**	–
**16**	–
Ningnanmycin	85.9 ± 4.6
Ribavirin	75.9 ± 3.5

^a^ Compounds obtained and the commercial antiviral agents used for control were tested for their antiviral activities in a concentration of 0.5 mM. – = no inhibitory effect observed.

**Table 2:** NMR data of Compounds **1** and **2**.

Position	1 ^a^		2 ^b^	
	δ_H_, Mult. (*J* in Hz)	δ_C_ (in ppm) ^c^	δ_H_, Mult. (*J* in Hz)	δ_C_ (in ppm)
1	3.69, d (8.1)	79.1, CH	3.68, d (8.0)	82.5, CH
2	3.95–3.87, overlap	82.9, CH	4.19, m	84.1 CH
3	5.65, m	124.2, CH	5.68, td (2.9, 1.5)	124.9, CH
4	–	134.6, C	–	137.4, C
5	2.24, br d (12.5)	39.6, CH	2.36, br d (13.2)	42.4, CH
6	1.94, td (14.8, 2.6) 1.86, dt (14.8, 2.9)	24.9, CH_2_	2.07, dt (14.9, 2.8) 1.97, ddd (14.9, 13.3, 2.6)	26.4, CH_2_
7	4.58, t (2.8)	77.0, CH	4.56, d (2.7)	80.4, CH
8	–	45.6, C	–	46.5, C
9	1.99, s	49.8, CH	2.67, s	45.2, CH
10	–	41.4, C	–	42.4, C
11	–	106.9, C	–	110.2, C
12	–	207.7, C	3.89, s	81.0, CH
13	3.21–3.08, overlap	39.5, CH	–	146.9, C
14	2.59, ddd (12.4, 8.5, 7.0)	42.3, CH	2.80, dd (13.5, 5.4)	48.0, CH
15	2.36–2.30, overlap	28.0, CH_2_	3.03, dd (18.7, 13.6) 2.64, dd (18.7, 5.4)	35.4, CH_2_
16	–	169.1, C	–	172.6, C
18	1.61, s	21.0, CH_3_	1.71, s	21.4, CH_3_
19	1.17, s	9.9, CH_3_	1.27, s	10.3, CH_3_
20a 20b	4.16, d (8.9) 3.90, d (8.9)	72.0, CH_2_	4.00, d (8.4) 3.48, d (8.4)	73.2, CH_2_
21	0.86, d (6.7)	10.0, CH_3_	5.22, s	120.3, CH_2_
Glc-1′	4.31, d (7.7)	105.2, CH	4.60, d (7.8)	105.0, CH
2′	2.95, m	74.2, CH	3.46–3.41, overlap	75.0, CH
3′	3.21–3.08, overlap	76.3, CH	3.58, t, (8.9)	88.1, CH
4′	3.02, td, (9.3, 4.1)	70.0, CH	3.46–3.41, overlap	69.9, CH
5′	3.21–3.08, overlap	76.7, CH	3.31–3.25, overlap	78.2, CH
6′	3.62–3.70, overlap 3.43, dt (11.7, 5.9)	61.1, CH_2_	3.89, dd (11.8, 2.3) 3.71, dd (12.0, 5.4)	62.6, CH_2_
1″	–	–	4.56, d (7.9)	105.2, CH
2″	–	–	3.31–3.25, overlap	75.5, CH
3″	–	–	3.39, t, (9.0)	77.8, CH
4″	–	–	3.31–3.25, overlap	71.6, CH
5″	–	–	3.37–3.33, overlap	77.6, CH
6″	–	–	3.89, dd (11.8, 2.3) 3.64, dd (11.9, 6.3)	62.6, CH_2_

^a 13^C-NMR spectroscopic data (δ) measured in dimethyl sulfoxide-*d*_6_ at 125 MHz and referenced to the solvent residual peak at δ 39.52, ^1^H-NMR spectroscopic data measured in dimethyl sulfoxide-*d*_6_ at 500 MHz and referenced to the solvent residual peak at δ 3.33. ^b 13^C-NMR spectroscopic data (δ) measured in methanol-*d*_4_ at 125 MHz and referenced to the solvent residual peak at δ 49.00, ^1^H-NMR spectroscopic data measured in methanol-*d*_4_ at 500 MHz and referenced to the solvent residual peak at δ 4.87. ^c^ Assignments of chemical shifts are based on the analysis of one- and two-dimensional NMR spectra. CH_3_, CH_2_, CH and C multiplicities were determined by DEPT and HSQC experiments.

## Supplementary Materials

The following are available online, HRESIMS, IR, ^1^H- and ^13^C-, DEPT, COSY, HSQC, HMBC, and NOESY NMR spectra of compounds **1** and **2** (Figures S1–S18); ^1^H- and ^13^C-NMR spectra of **3**–**16** (Figures S19–S46).

## References

[1] Wang Y., Wang W.J., Su C., Zhang D.M., Xu L.P., He R.R., Wang L., Zhang J., Zhang X.Q., Ye W.C. (2013). Cytotoxic quassinoids from *Ailanthus altissima*. Bioorg. Med. Chem. Lett..

[2] De Feo V., De Martino L., Quaranta E., Pizza C. (2003). Isolation of phytotoxic compounds from tree-of-heaven (*Ailanthus altissima* Swingle). J. Agric. Food. Chem..

[3] Nanjing University of Chinese Medicine (2006). Dictionary of Traditional Chinese Materia Medica.

[4] Bai W., Yang H.Y., Jiao X.Z., Feng K.N., Chen J.J., Gao K. (2018). Structurally diverse highly oxygenated triterpenoids from the roots of *Ailanthus altissima* and their cytotoxicity. J. Nat. Prod..

[5] Ni J.C., Shi J.C., Tan Q.W., Chen Q.J. (2019). Two new compounds from the samara of *Ailanthus altissima*. Nat. Prod. Res..

[6] Alves I.A.B.S., Miranda H.M., Soares L.A.L., Randau K.P. (2014). Simaroubaceae family: Botany, chemical composition and biological activities. Rev. Bras. Farmacogn..

[7] Ni J.C., Shi J.T., Tan Q.W., Chen Q.J. (2017). Phenylpropionamides, piperidine, and phenolic derivatives from the samara of *Ailanthus altissima*. Molecules.

[8] Tamura S., Fukamiya N., Okano M., Koyama J., Koike K., Tokuda H., Aoi W., Takayasu J., Kuchide M., Nishino H. (2003). Three new quassinoids, ailantinol E, F, and G, from *Ailanthus altissima*. Chem. Pharm. Bull..

[9] Kim H.M., Kim S.J., Kim H.Y., Ryu B., Kwak H., Hur J., Choi J.H., Jang D.S. (2015). Constituents of the stem barks of *Ailanthus altissima* and their potential to inhibit LPS-induced nitric oxide production. Bioorg. Med. Chem. Lett..

[10] Hong Z.L., Xiong J., Wu S.B., Zhu J.J., Hong J.L., Zhao Y., Xia G., Hu J.F. (2013). Tetracyclic triterpenoids and terpenylated coumarins from the bark of *Ailanthus altissima* (“Tree of heaven”). Phytochemistry.

[11] Tan Q.W., Ouyang M.A., Wu Z.J. (2012). A new seco-neolignan glycoside from the root bark of *Ailanthus altissima*. Nat. Prod. Res..

[12] Tan Q.W., Wu Z.J., Ouyang M.A. (2008). Research progress in chemical constituents and bioactivities of *Ailanthus ailanthus*. Nat. Prod. Res. Dev..

[13] Tan Q.W., Ni J.C., Zheng L.P., Fang P.H., Shi J.T., Chen Q.J. (2018). Anti-Tobacco mosaic virus quassinoids from *Ailanthus altissima* (Mill.) Swingle. J. Agric. Food Chem..

[14] Qiao L.R., Yang L., Zhang D.W., Zou J.H., Dai J.G. (2011). Studies on chemical constitutes from callus cultures of *Stellera chamaejasme*. China J. Chin. Mater. Med..

[15] Xie L.H., Akao T., Hamasaki K., Deyama T., Hattori M. (2003). Biotransformation of pinoresinol diglucoside to mammalian lignans by human intestinal microflora, and isolation of *Enterococcus faecalis* strain PDG-1 responsible for the transformation of (+)-pinoresinol to (+)-lariciresinol. Chem. Pharm. Bull..

[16] Chang C.I., Hsu C.M., Li T.S., Huang S.D., Lin C.C., Yen C.H., Chou C.H., Cheng H.L. (2014). Constituents of the stem of *Cucurbita moschata* exhibit antidiabetic activities through multiple mechanisms. J. Funct. Foods.

[17] Carpinella M.C., Giorda L.M., Ferrayoli C.G., Palacios S.M. (2003). Antifungal effects of different organic extracts from M*elia azedarach* L. on phytopathogenic fungi and their isolated active components. J. Agric. Food Chem..

[18] Jutiviboonsuk A., Zhang H., Tan G.T., Ma C., Hung N.V., Cuong N.M., Bunyapraphatsara N., Soejarto D.D., Fong H.H.S. (2005). Bioactive constituents from roots of *Bursera tonkinensis*. Phytochemistry.

[19] Zhong X.N., Ide T., Otsuka H., Hirata E., Takeda Y. (1998). (+)-Isolarisiresinol 3α-*O*-sulphate from leaves of *Myrsine seguinii*. Phytochemistry.

[20] Chu H.B., Zeng G.Z., Zhu M.J., He W.J., Zhang Y.M., Tan N.H. (2011). Chemical Constituents of *Pedicularis densispica* Franch. Z. Für Naturforsch. B.

[21] Li L., Seeram N.P. (2010). Maple syrup phytochemicals include lignans, coumarins, a stilbene, and other previously unreported antioxidant phenolic compounds. J. Agric. Food Chem..

[22] Xu J.J., Tan N.H. (2012). New lignans from *Jatropha curcas* Linn. Z. Für Naturforsch. B.

[23] Li S., Lundquist K., Wallis A.F.A. (1998). Revised structure for a neolignan from *Brucea javanica*. Phytochemistry.

[24] Matsuda N., Kikuchi M. (1996). Studies on the constituents of *Lonicera* species. X. Neolignan glycosides from the leaves of *Lonicera gracilipes* var. glandulosa Maxim. Chem. Pharm. Bull..

[25] Hsiao J.J., Chiang H.C. (1995). Lignans from the wood of *Aralia bipinnata*. Phytochemistry.

[26] Lehmann S., Herrmann F., Kleemann K., Spiegler V., Liebau E., Hensel A. (2020). Extract and the quassinoid ailanthone from *Ailanthus altissima* inhibit nematode reproduction by damaging germ cells and rachis in the model organism *Caenorhabditis elegans*. Fitoterapia.

[27] Vieira I.J.C., Braz-Filho R. (2006). Quassinoids: Structural diversity, biological activity and synthetic studies. Stud. Nat. Prod. Chem..

[28] Li Z., Ruan J.Y., Sun F., Yan J.J., Wang J.L., Zhang Z.X., Zhang Y., Wang T. (2019). Relationship between structural characteristics and plant sources along with pharmacology research of quassinoids. Chem. Pharm. Bull..

[29] Bailly C. (2020). Anticancer properties and mechanism of action of the quassinoid ailanthone. Phytother. Res..

[30] Ding H., Yu X., Hang C., Gao K., Lao X., Jiao Y., Yan Z. (2020). Ailanthone: A novel potential drug for treating human cancer. Oncol. Lett..

[31] Xie L.H., Lin Q.Y. (2011). Plant Virology.

[32] Wu J., Song B. (2016). Research progress of anti-virus agents for plants in China. Sci. Sin. Chim..

[33] Long C.W., Li P., Chen M.H., Dong L.R., Hu D.Y., Song B.A. (2015). Synthesis, anti-Tobacco mosaic virus and Cucumber mosaic virus activity, and 3D-QSAR study of novel 1,4-pentadien-3-one derivatives containing 4-thioquinazoline moiety. Eur. J. Med. Chem..

[34] Han G., Chen L., Wang Q., Wu M., Liu Y., Wang Q. (2018). Design, synthesis, and anti-Tobacco mosaic virus activity of water-soluble chiral quaternary ammonium salts of phenanthroindolizidines alkaloids. J. Agric. Food Chem..

[35] Han Y., Luo Y., Qin S., Xi L., Wan B., Du L. (2014). Induction of systemic resistance against Tobacco mosaic virus by ningnanmycin in tobacco. Pestic. Biochem. Physiol..

[36] Li X., Chen K., Gao D., Wang D., Huang M., Zhu H., Kang J. (2018). Binding studies between cytosinpeptidemycin and the superfamily 1 helicase protein of Tobacco mosaic virus. RSC Adv..

[37] Li X., Hao G., Wang Q., Chen Z., Ding Y., Yu L., Hu D., Song B. (2017). Ningnanmycin inhibits Tobacco mosaic virus virulence by binding directly to its coat protein discs. Oncotarget.

[38] Shen J.G., Zhang Z.K., Wu Z.J., Ouyang M.A., Xie L.H., Lin Q.Y. (2008). Antiphytoviral activity of bruceine-d from *Brucea javanica* seeds. Pest. Manag. Sci..

[39] Wu Z.J., Ouyang M.A., Wang C.Z., Zhang Z.K., Shen J.G. (2007). Anti-Tobacco mosaic virus (TMV) triterpenoid saponins from the leaves of *Ilex oblonga*. J. Agric. Food Chem..

