# Supplementary material for: Two Novel Quassinoid Glycosides with Antiviral Activity from the Samara of Ailanthus altissima
Source: Molecules. 2020 Dec 2;25(23):5679. doi: 10.3390/molecules25235679 (PMC7730543; doi:10.3390/molecules25235679)
Supplement: Supplementary file 1 [file molecules-25-05679-s001.pdf]

Supplementary Materials

Title: Two Novel Quassinoid Glycosides with Antiviral Activity from the Samara of *Ailanthus altissima*

Authors: Qing-Wei Tan [1,*,†], Jian-Cheng Ni [2,†], Jian-Ting Shi [1], Jian-Xuan Zhu [1] and Qi-Jian Chen [1,*]

S1. HRESIMS spectra of chuglycoside J (**1**).

S2. IR spectra of chuglycoside J (**1**).

S3. ^{1}H-NMR spectra of chuglycoside J (**1**) (500 MHz, Dimethyl sulfoxide-d_6).

S4. ^{13}C NMR spectra of chuglycoside J (**1**) (125 MHz, Dimethyl sulfoxide-d_6).

S5. DEPT of chuglycoside J (**1**).

S6. ^{1}H-^{1}H COSY of chuglycoside J (**1**).

S7. HSQC of chuglycoside J (**1**).

S8. HMBC of chuglycoside J (**1**).

S9. NOESY of chuglycoside J (**1**).

S10. HRESIMS spectra of chuglycoside K (**2**).

S11. IR spectra of chuglycoside K (**2**).

S12. ^{1}H-NMR spectra of chuglycoside K (**2**) (500 MHz, Methanol-d_4).

S13. ^{13}C NMR spectra of chuglycoside K (**2**) (125 MHz, Methanol-d_4).

S14. DEPT of chuglycoside K (**2**).

S15. ^{1}H-^{1}H COSY of chuglycoside K (**2**).

S16. HSQC of chuglycoside K (**2**).

S17. HMBC of chuglycoside K (**2**).

S18. NOESY of chuglycoside K (**2**).

S19. ^{1}H-NMR spectra of tetrahydro-2-(4-hydroxy-3-methoxyphenyl)-4-[(4-hydroxyphenyl) methyl]-3-furanmethanol (**3**) (500 MHz, Methanol-d_4).

S20. ^{13}C NMR spectra of tetrahydro-2-(4-hydroxy-3-methoxyphenyl)-4-[(4-hydroxyphenyl) methyl]-3-furanmethanol (**3**) (125 MHz, Methanol-d_4).

S21. ^{1}H-NMR spectra of (+)-lariciresinol (**4**) (500 MHz, Methanol-d_4).

S22. ^{13}C NMR spectra of (+)-lariciresinol (**4**) (125 MHz, Methanol-d_4).

S23. ^{1}H-NMR spectra of (+)-(1*R*,2*S*,5*R*,6*S*)-2,6-di(4'-hydroxyphenyl)-3,7-dioxabicyclo[3.3.0]octane (**5**) (500 MHz, Methanol-d_4).

S24. ^{13}C NMR spectra of (+)-(1*R*,2*S*,5*R*,6*S*)-2,6-di(4'-hydroxyphenyl)-3,7-dioxabicyclo[3.3.0]octane (**5**) (125 MHz, Methanol-d_4).

S25. ^{1}H-NMR spectra of (+)-pinoresinol (**6**) (500 MHz, Methanol-d_4).

S26. ^{13}C NMR spectra of (+)-pinoresinol (**6**) (125 MHz, Methanol-d_4).

S27. ^{1}H-NMR spectra of (+)-isolariciresinol (**7**) (500 MHz, Methanol-d_4).

S28. ^{13}C NMR spectra of (+)-isolariciresinol (**7**) (125 MHz, Methanol-d_4).

S29. ^{1}H-NMR spectra of (+)-isolariciresinol 3*α*-*O*-*β*-glucopyranoside (**8**) (500 MHz, Methanol-d_4).

S30. ^{13}C NMR spectra of (+)-isolariciresinol 3*α*-*O*-*β*-glucopyranoside (**8**) (125 MHz, Methanol-d_4).

S31. ^{1}H-NMR spectra of burselignan (**9**) (500 MHz, Methanol-d_4).

S32. ^{13}C NMR spectra of burselignan (**9**) (125 MHz, Methanol-d_4).

S33. ^{1}H-NMR spectra of densispicoside (**10**) (500 MHz, Methanol-d_4).

S34. ^{13}C NMR spectra of densispicoside (**10**) (125 MHz, Methanol-d_4).

S35. ^{1}H-NMR spectra of secoisolariciresinol (**11**) (500 MHz, Methanol-d_4).

S36. ^{13}C NMR spectra of secoisolariciresinol (**11**) (125 MHz, Methanol-d_4).

S37. ^{1}H-NMR spectra of dehydroconiferyl alcohol (**12**) (500 MHz, Methanol-d_4).

S38. ^{13}C NMR spectra of dehydroconiferyl alcohol (**12**) (125 MHz, Methanol-d_4).

S39. ^{1}H-NMR spectra of curcasinlignan B (**13**) (500 MHz, Methanol-d_4).

S40. ^{13}C NMR spectra of curcasinlignan B (**13**) (125 MHz, Methanol-d_4).

S41. ^{1}H-NMR spectra of *erythro*-guaiacylglycerol-*β*-*O*-4′-coniferyl ether (**14**) (500 MHz, Methanol-d_4).

S42. ^{13}C NMR spectra of *erythro*-guaiacylglycerol-*β*-*O*-4′-coniferyl ether (**14**) (125 MHz, Methanol-d_4).

S43. ^{1}H-NMR spectra of 7*R*,8*R*-*threo*-4,7,9,9'-tetrahydroxy-3,3'-dimethoxy-8-*O*-4'-neolignan (**15**) (500 MHz, Methanol-d_4).

S44. ^{13}C NMR spectra of 7*R*,8*R*-*threo*-4,7,9,9'-tetrahydroxy-3,3'-dimethoxy-8-*O*-4'-neolignan (**15**) (125 MHz, Methanol-d_4).

S45. ^{1}H-NMR spectra of *threo*-2,3-*bis*-(4-hydroxy-3-methoxyphenyl)-3-methoxypropanol (**16**) (500 MHz, Methanol-d_4).

S46. ^{13}C NMR spectra of *threo*-2,3-*bis*-(4-hydroxy-3-methoxyphenyl)-3-methoxypropanol (**16**) (125 MHz, Methanol-d_4).

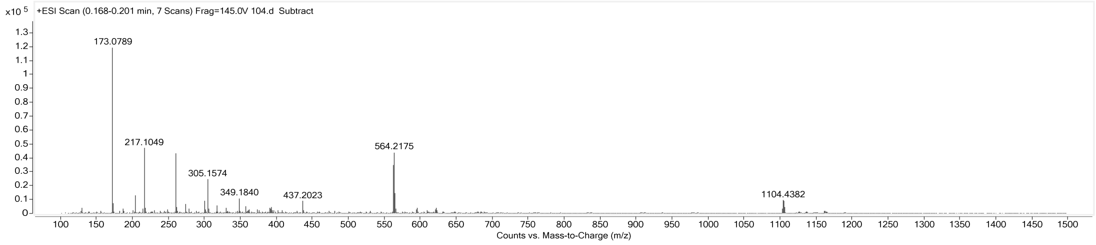

S1. HRESIMS spectra of chuglycoside J (**1**).

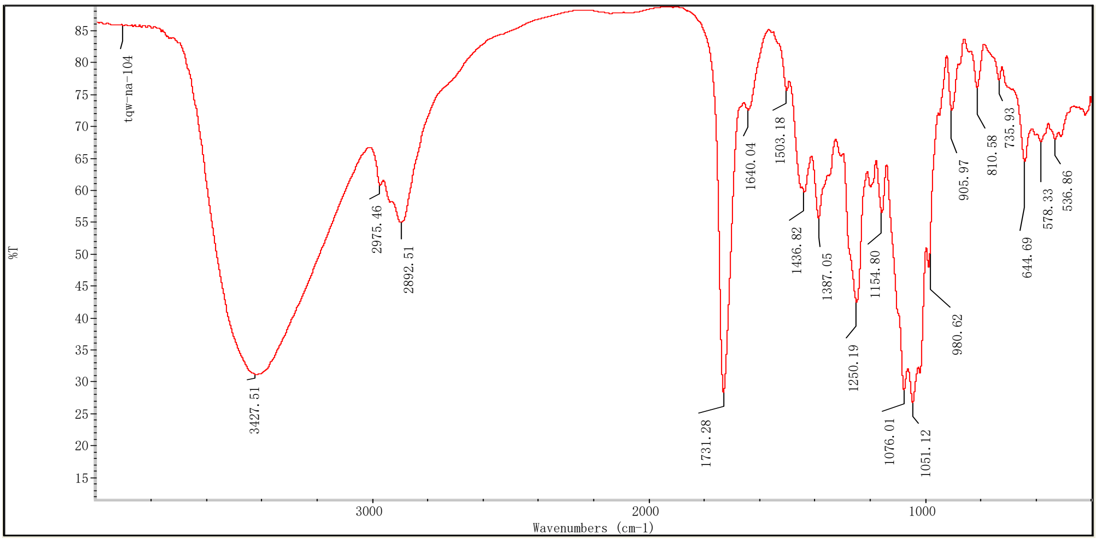

S2. IR spectra of chuglycoside J (**1**).

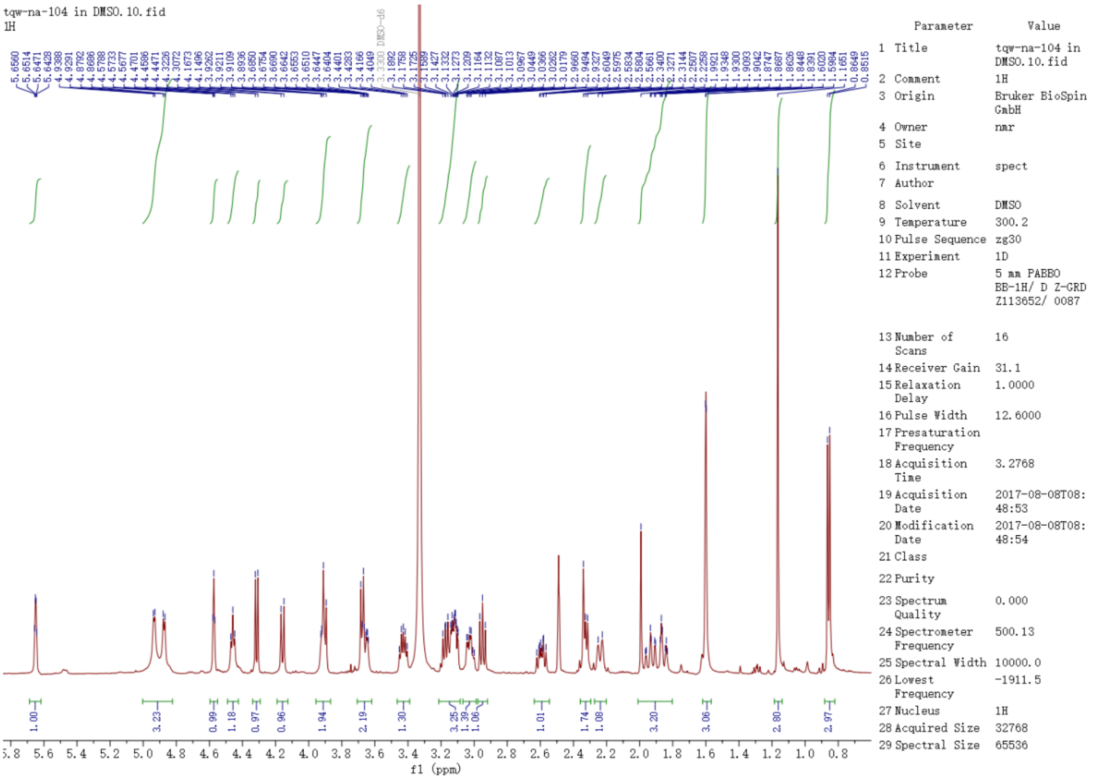

S3. ^{1}H-NMR spectra of chuglycoside J (**1**) (500 MHz, Dimethyl sulfoxide-*d*$_6$).

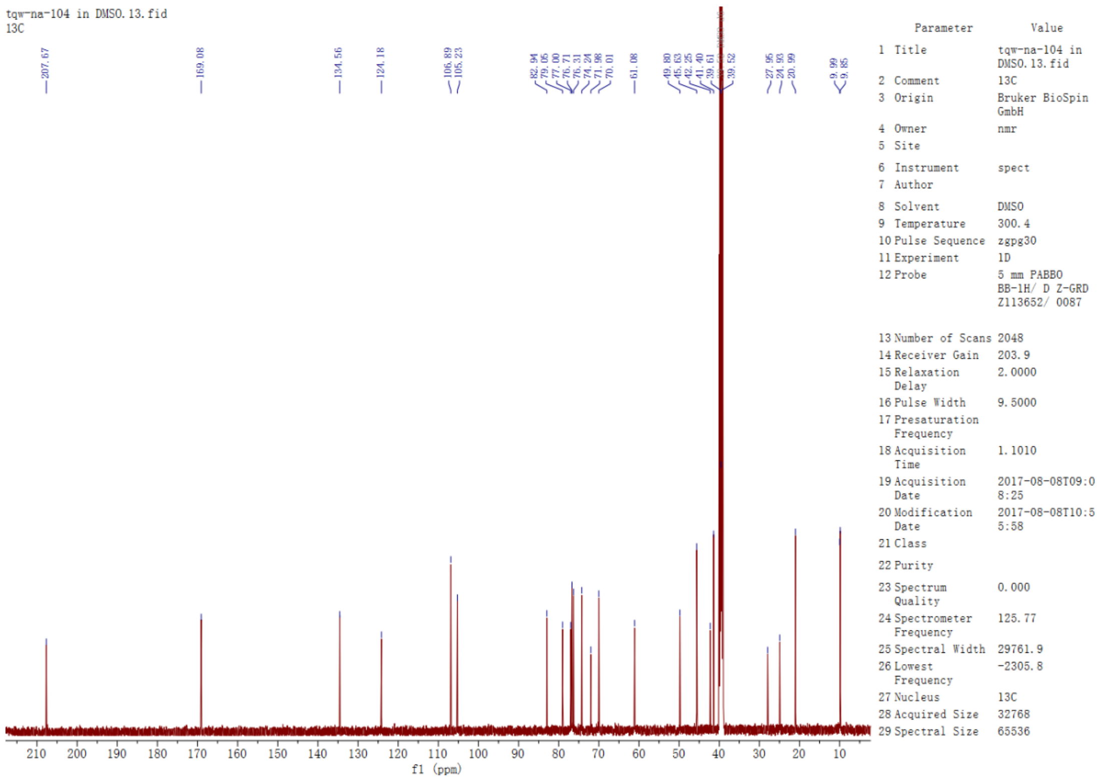

S4. ^{13}C NMR spectra of chuglycoside J (**1**) (125 MHz, Dimethyl sulfoxide-d_6).

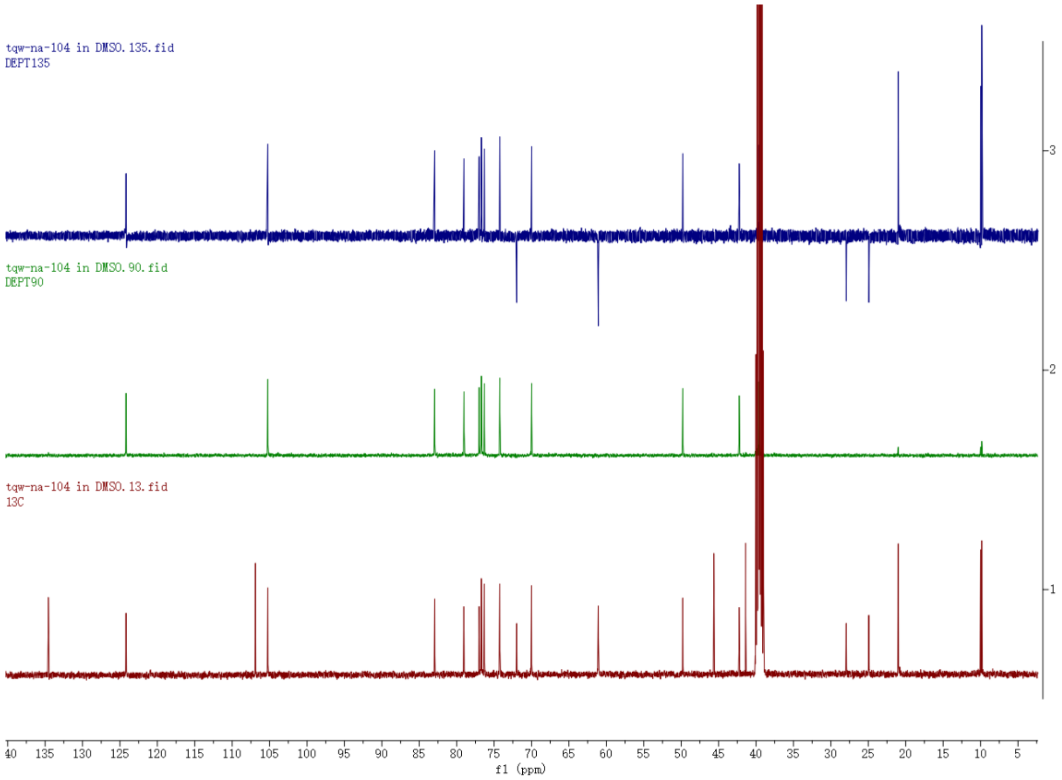

S5. DEPT of chuglycoside J (**1**).

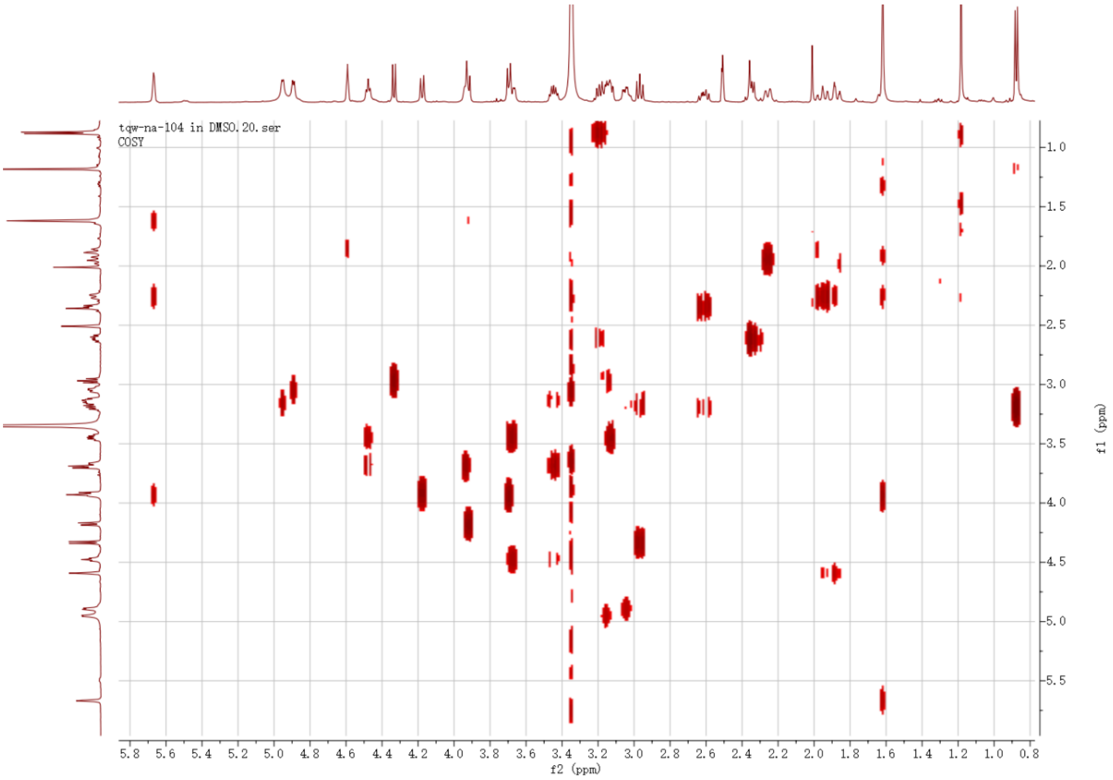

S6. ^{1}H-^{1}H COSY of chuglycoside J (**1**).

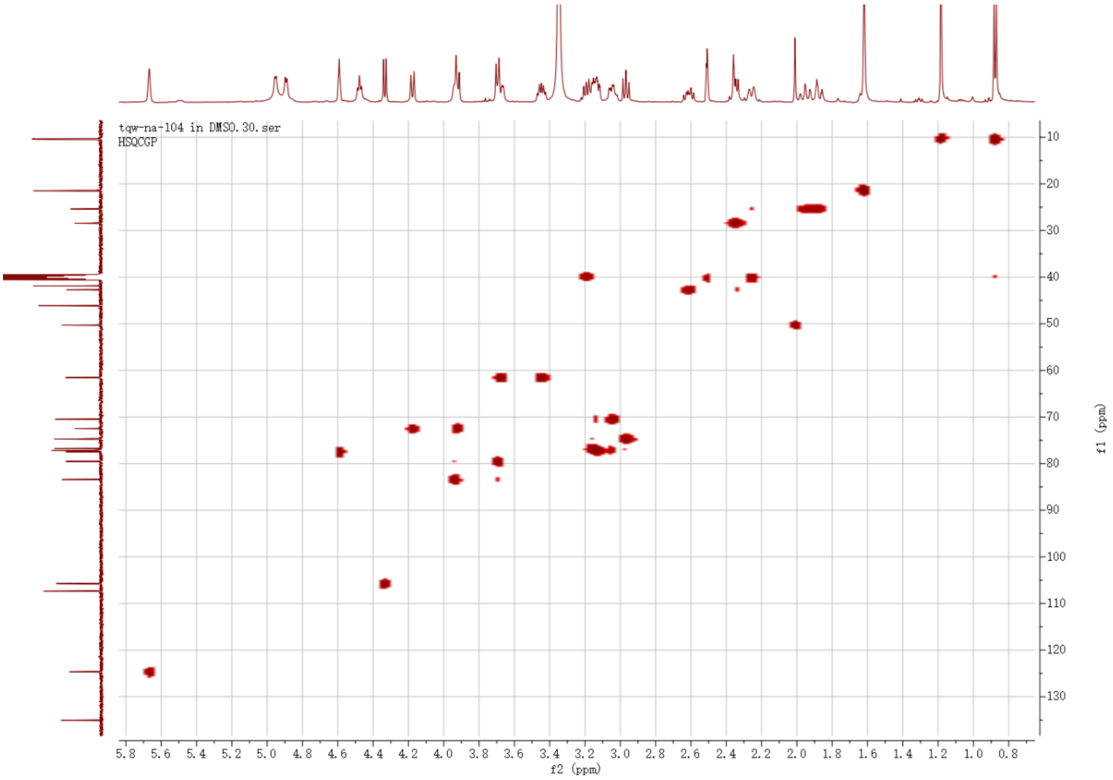

S7. HSQC of chuglycoside J (**1**).

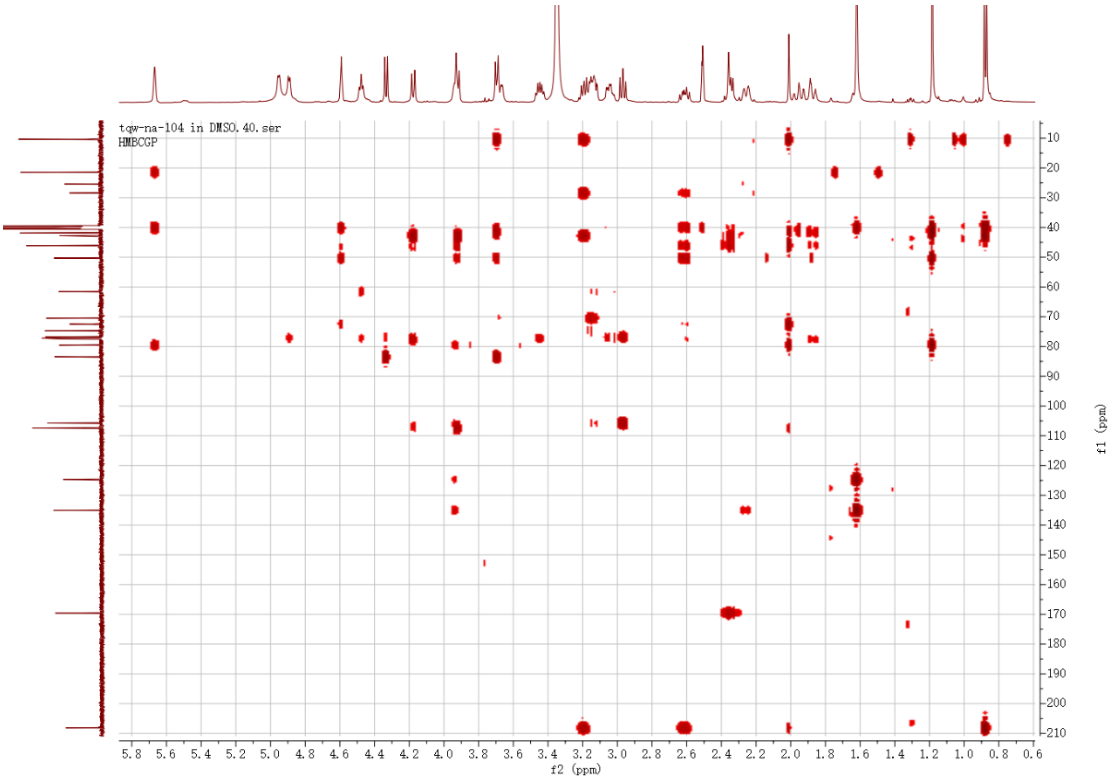

S8. HMBC of chuglycoside J (**1**).

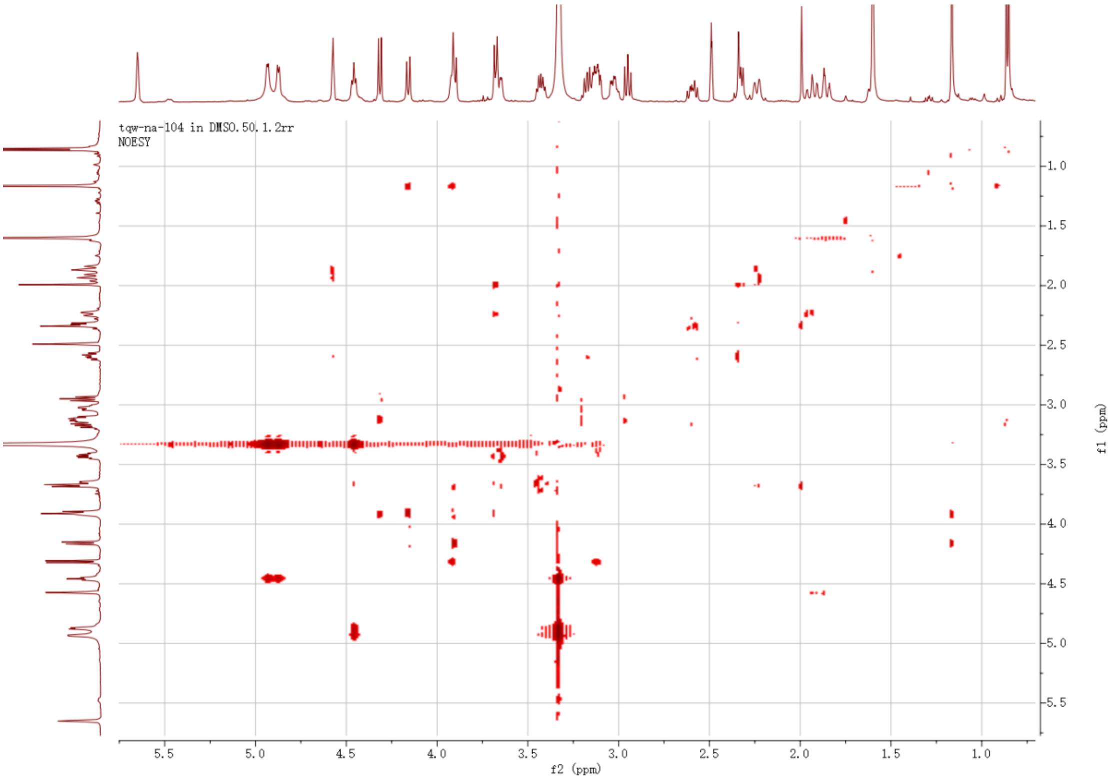

S9. NOESY of chuglycoside J (**1**).

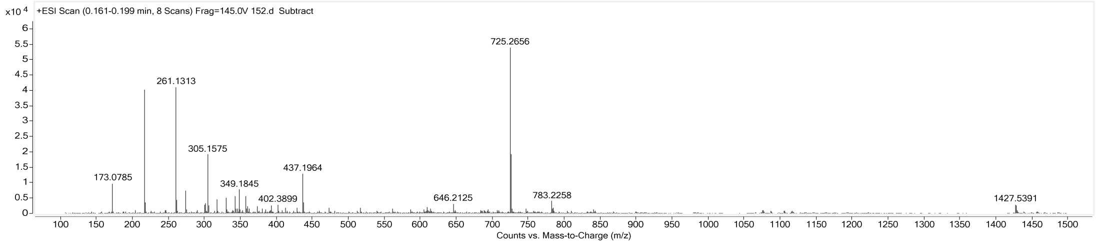

S10. HRESIMS spectra of chuglycoside K (**2**).

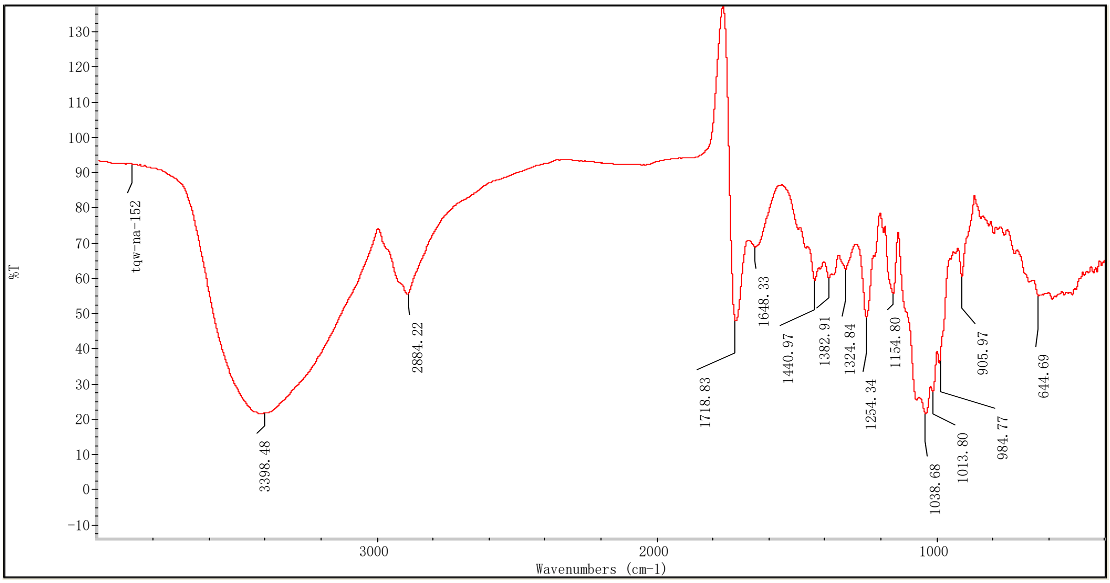

S11. IR spectra of chuglycoside K (**2**).

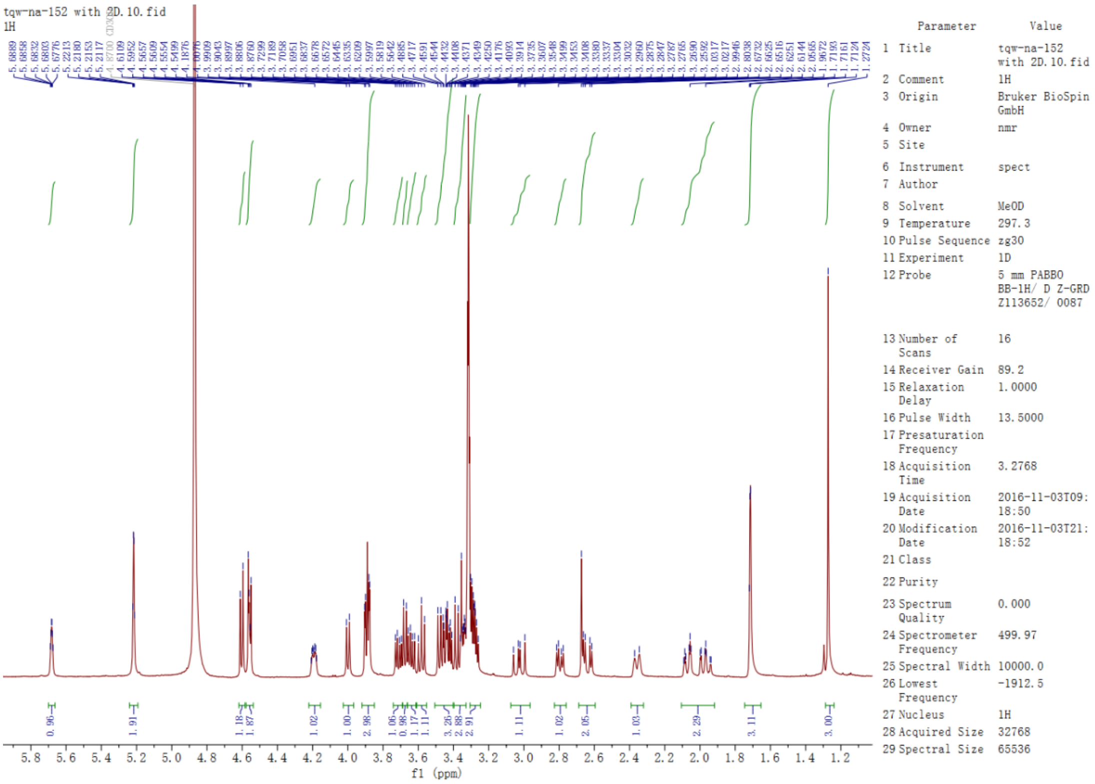

S12. ^{1}H-NMR spectra of chuglycoside K (**2**) (500 MHz, Methanol-d_4).

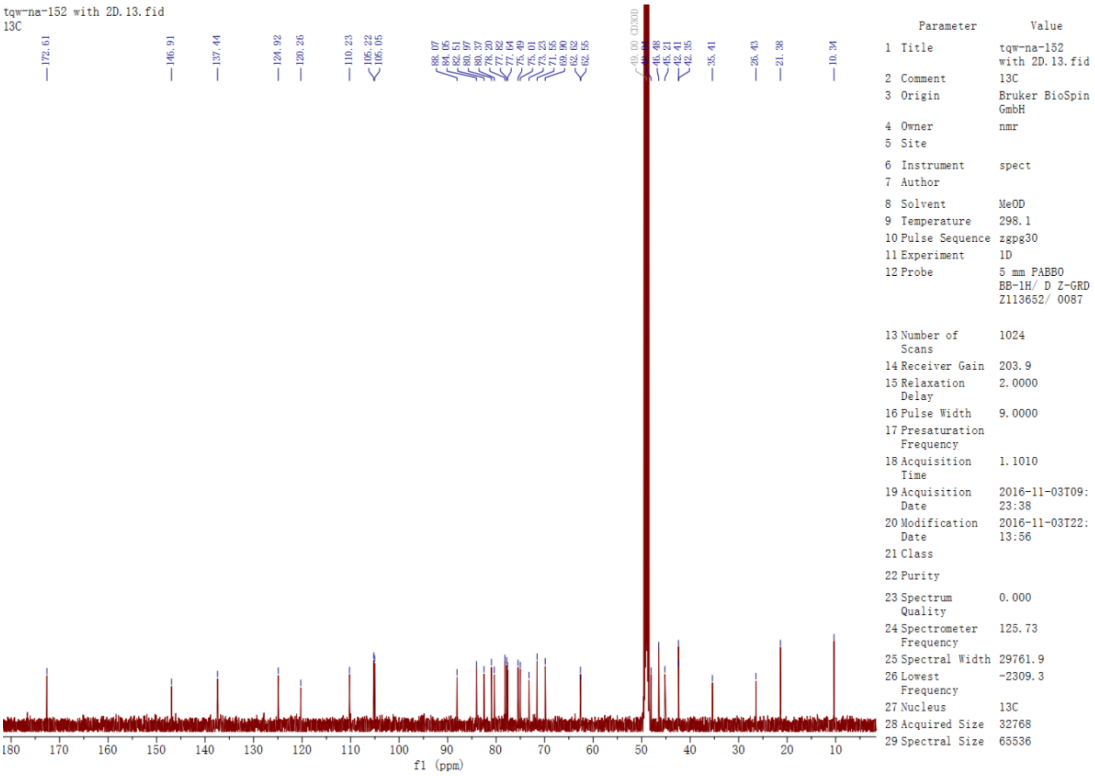

S13. ^{13}C NMR spectra of chuglycoside K (**2**) (125 MHz, Methanol-d_4).

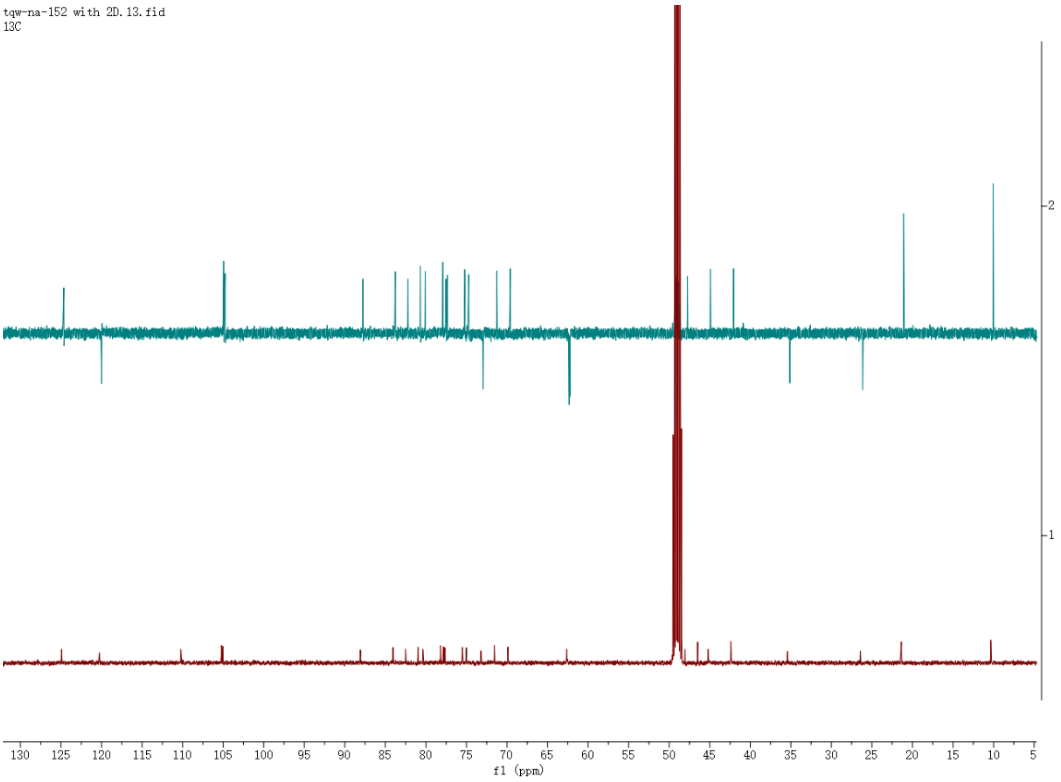

S14. DEPT of chuglycoside K (**2**).

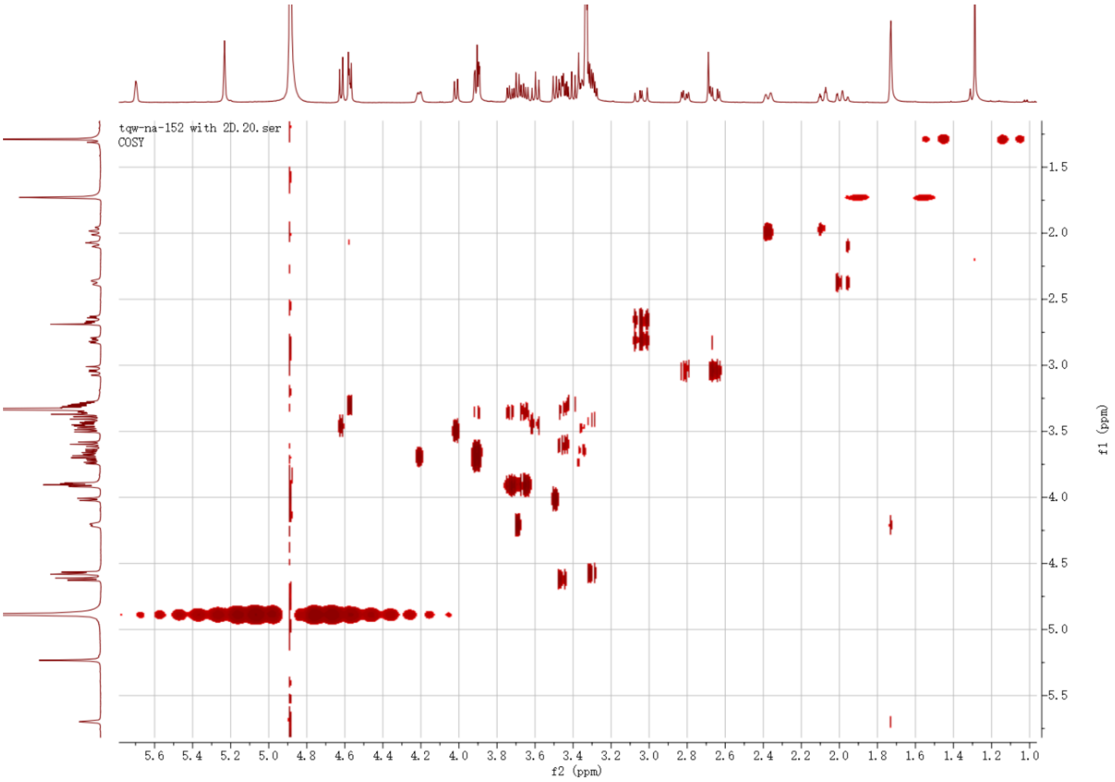

S15. ^{1}H-^{1}H COSY of chuglycoside K (**2**).

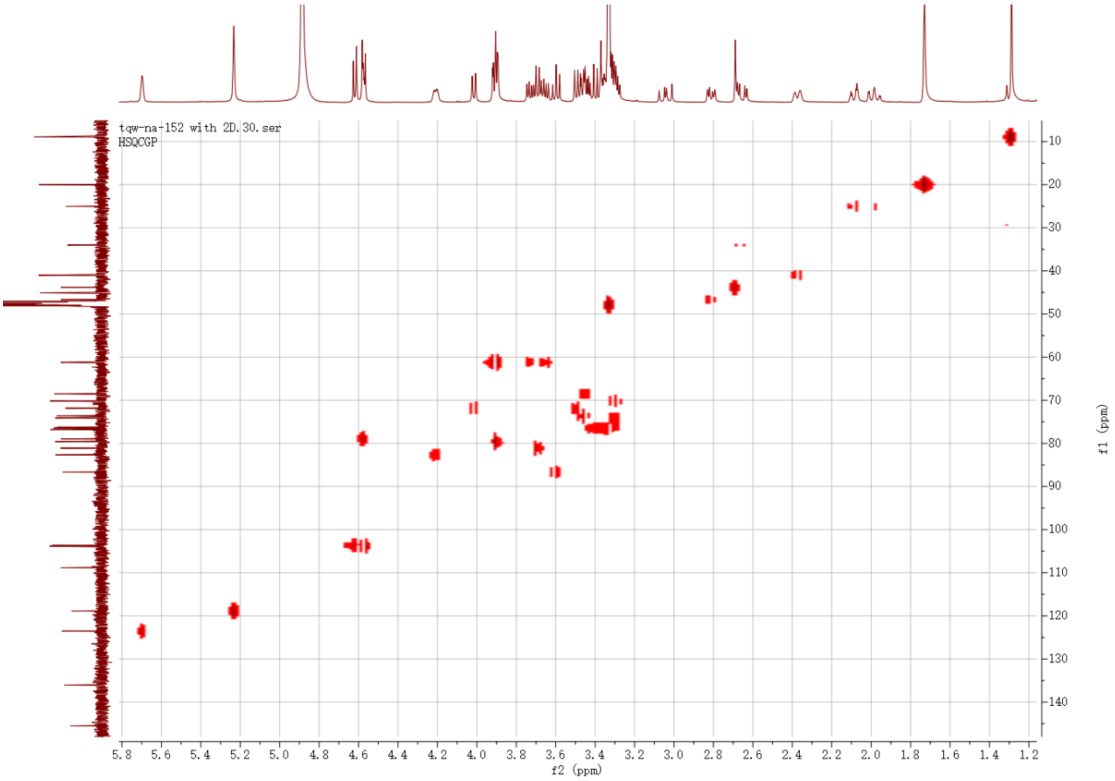

S16. HSQC of chuglycoside K (**2**).

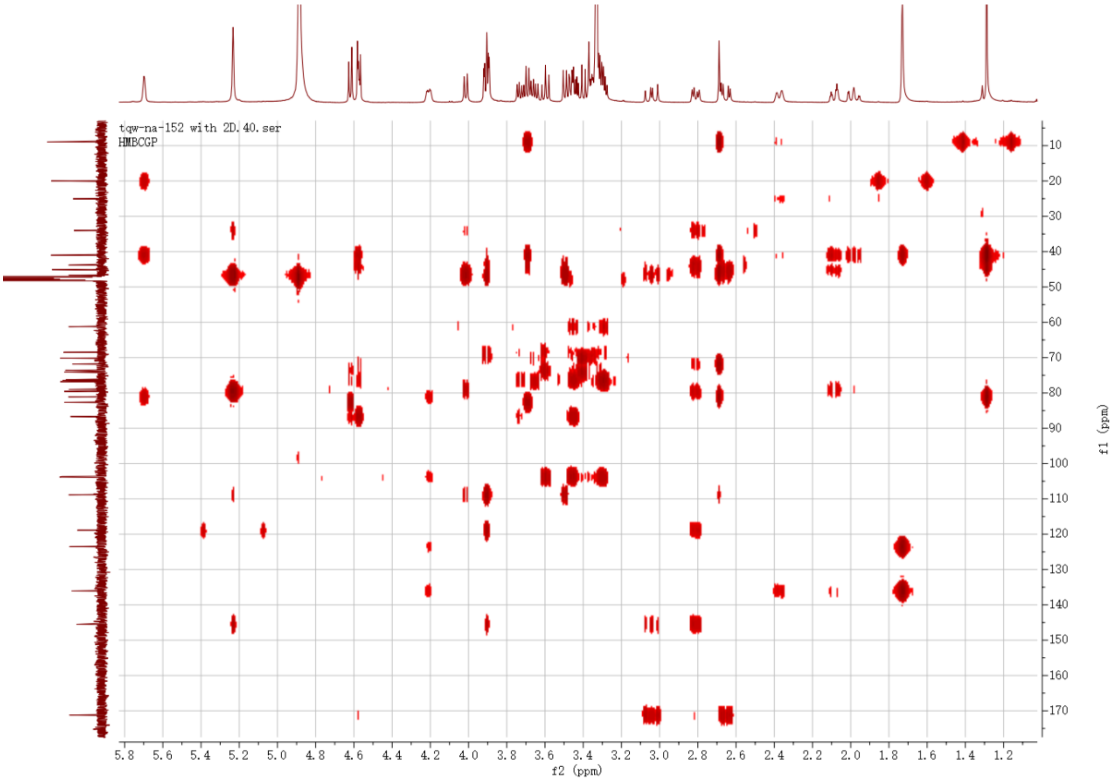

S17. HMBC of chuglycoside K (**2**).

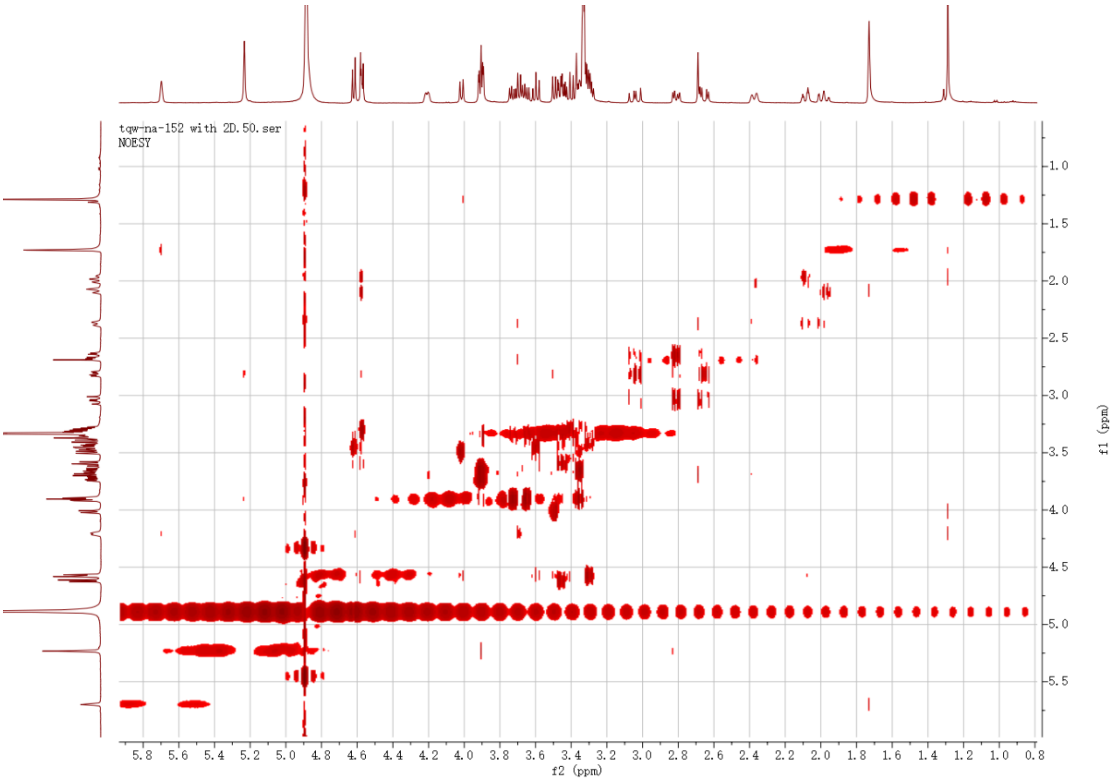

S18. NOESY of chuglycoside K (**2**).

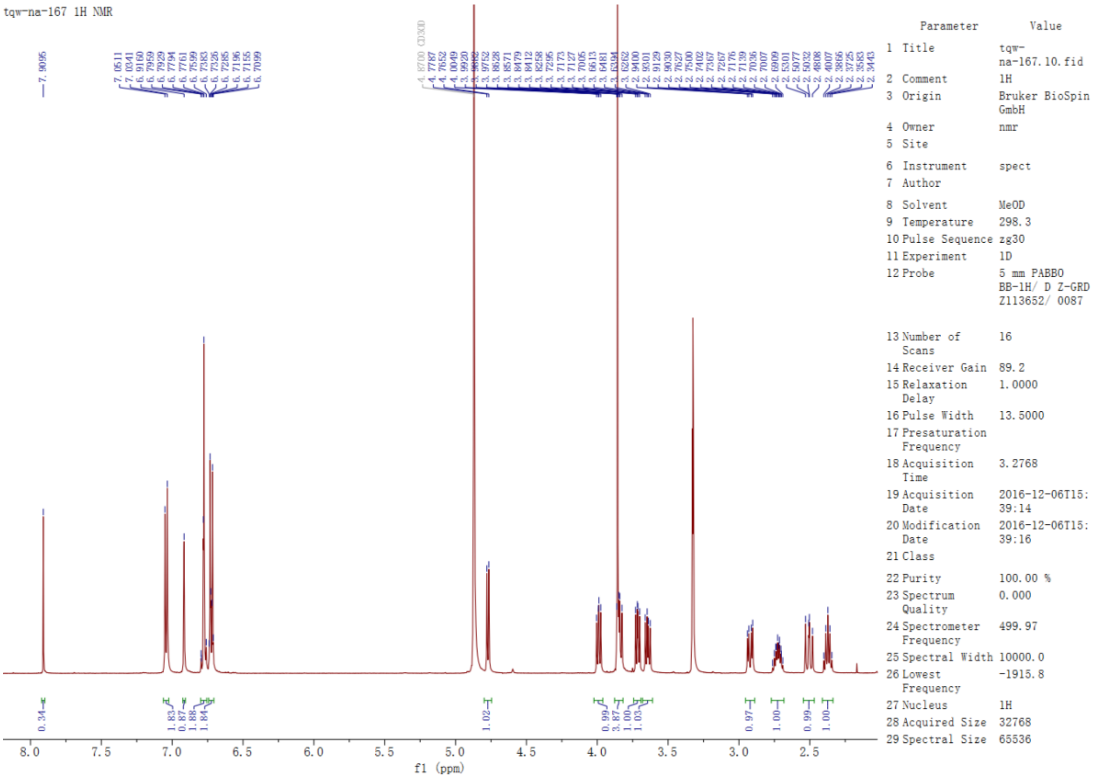

S19. ^{1}H-NMR spectra of tetrahydro-2-(4-hydroxy-3-methoxyphenyl)-4-[(4-hydroxyphenyl) methyl]-3-furanmethanol (**3**) (500 MHz, Methanol-*d*4).

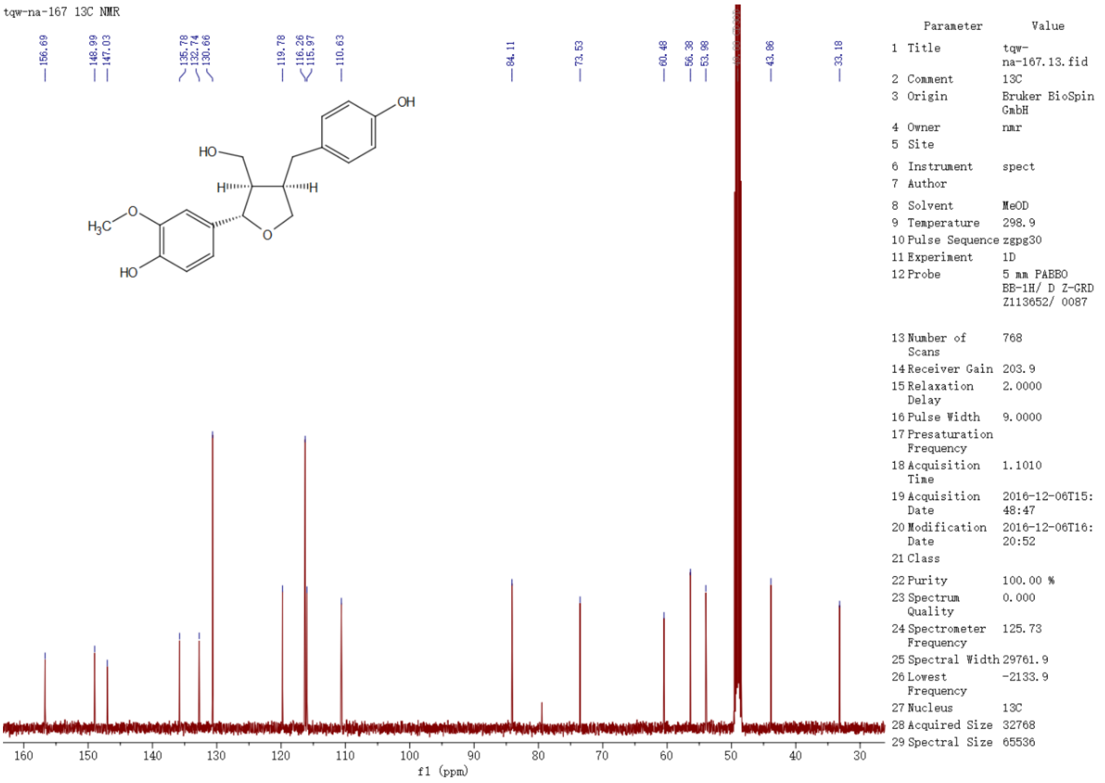

S20. ^{13}C NMR spectra of tetrahydro-2-(4-hydroxy-3-methoxyphenyl)-4-[(4-hydroxyphenyl) methyl]-3-furanmethanol (**3**) (125 MHz, Methanol-*d*4).

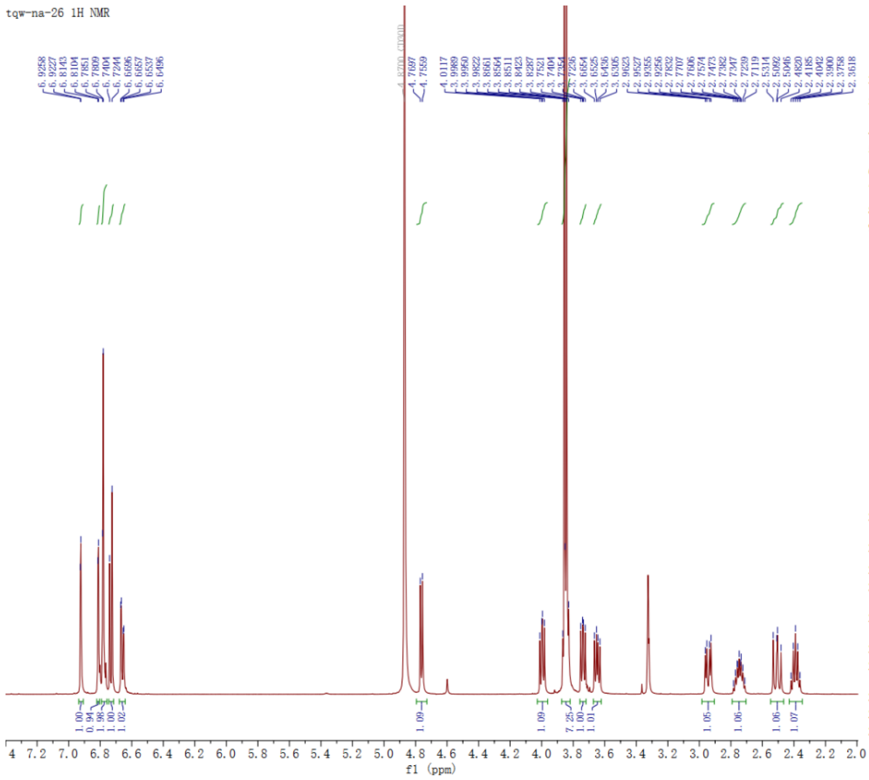

Parameter	Value
1 Title	tqw-na-26.10.1.1r
2 Comment	
3 Origin	Bruker BioSpin GmbH
4 Owner	nmr
5 Site	
6 Instrument	spect
7 Author	
8 Solvent	MeOD
9 Temperature	298.3
10 Pulse Sequence	zg30
11 Experiment	1D
12 Probe	5 mm PABBO BB-1H/ D Z-GRD Z113652/ 0087
13 Number of Scans	16
14 Receiver Gain	99.1
15 Relaxation Delay	1.0000
16 Pulse Width	11.0000
17 Presaturation Frequency	
18 Acquisition Time	3.2768
19 Acquisition Date	2015-05-14T10:34:47
20 Modification Date	2015-05-14T10:34:48
21 Class	
22 Purity	100.00 %
23 Spectrum Quality	0.000
24 Spectrometer Frequency	499.97
25 Spectral Width	10000.0
26 Lowest Frequency	-1915.5
27 Nucleus	1H
28 Acquired Size	32768
29 Spectral Size	65536

S21. ^{1}H-NMR spectra of (+)-lariciresinol (**4**) (500 MHz, Methanol-*d*$_4$).

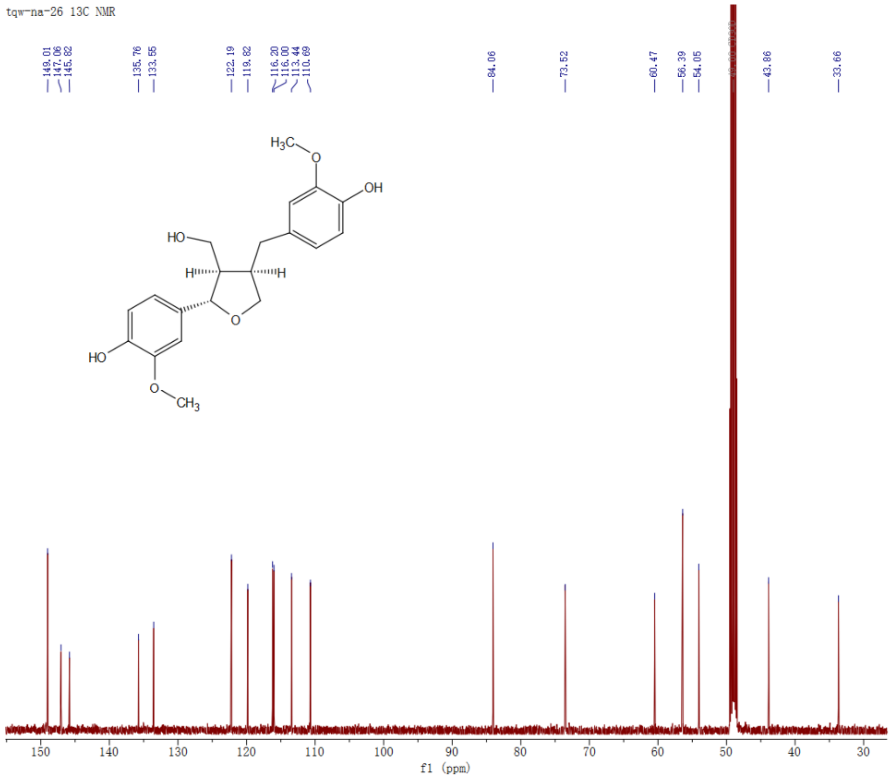

Parameter	Value
1 Title	tqw-na-26-13c.13.1.1r
2 Comment	
3 Origin	Bruker BioSpin GmbH
4 Owner	nmr
5 Site	
6 Instrument	spect
7 Author	
8 Solvent	MeOD
9 Temperature	300.2
10 Pulse Sequence	zgpg30
11 Experiment	1D
12 Probe	5 mm PABBO BB-1H/ D Z-GRD Z113652/ 0087
13 Number of Scans	640
14 Receiver Gain	203.9
15 Relaxation Delay	2.0000
16 Pulse Width	9.0000
17 Presaturation Frequency	
18 Acquisition Time	1.1010
19 Acquisition Date	2015-05-28T09:59:02
20 Modification Date	2015-05-28T10:32:13
21 Class	
22 Purity	100.00 %
23 Spectrum Quality	0.000
24 Spectrometer Frequency	125.72
25 Spectral Width	29761.9
26 Lowest Frequency	-2135.4
27 Nucleus	13C
28 Acquired Size	32768
29 Spectral Size	32768

S22. ^{13}C NMR spectra of (+)-lariciresinol (**4**) (125 MHz, Methanol-*d*$_4$).

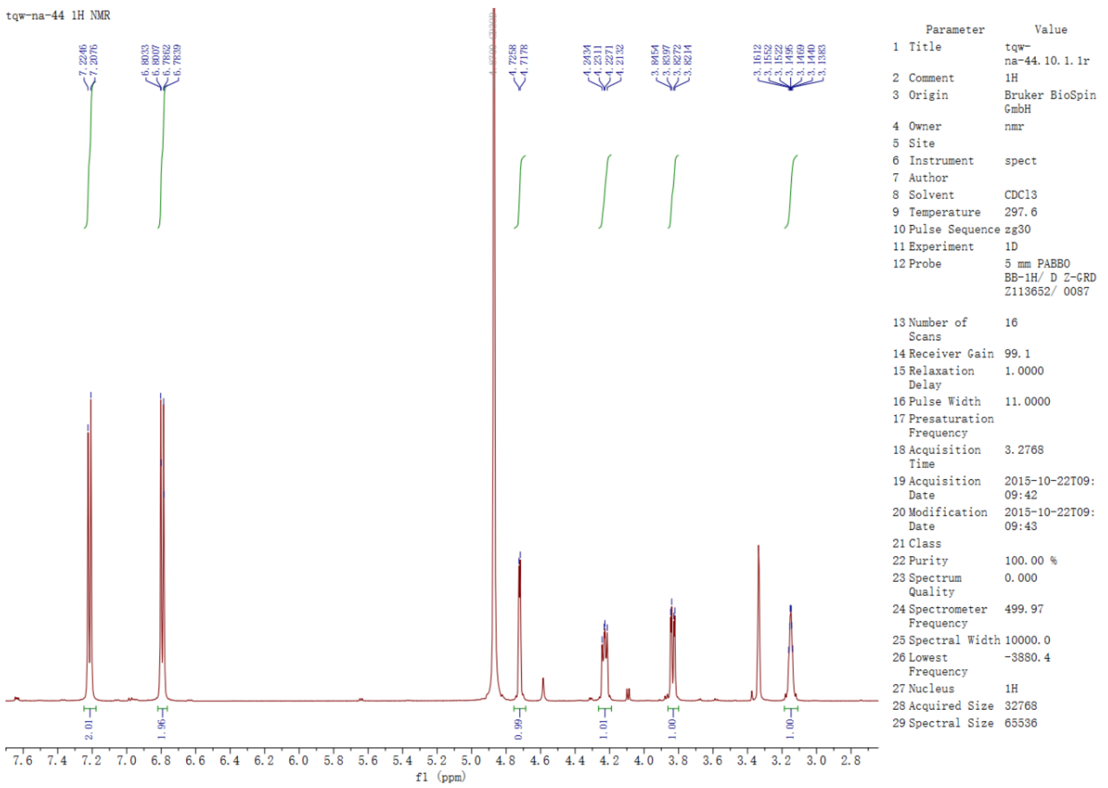

S23. ^{1}H-NMR spectra of (+)-(1*R*,2*S*,5*R*,6*S*)-2,6-di(4'-hydroxyphenyl)-3,7-dioxabicyclo[3.3.0]octane (**5**) (500 MHz, Chloroform-*d*).

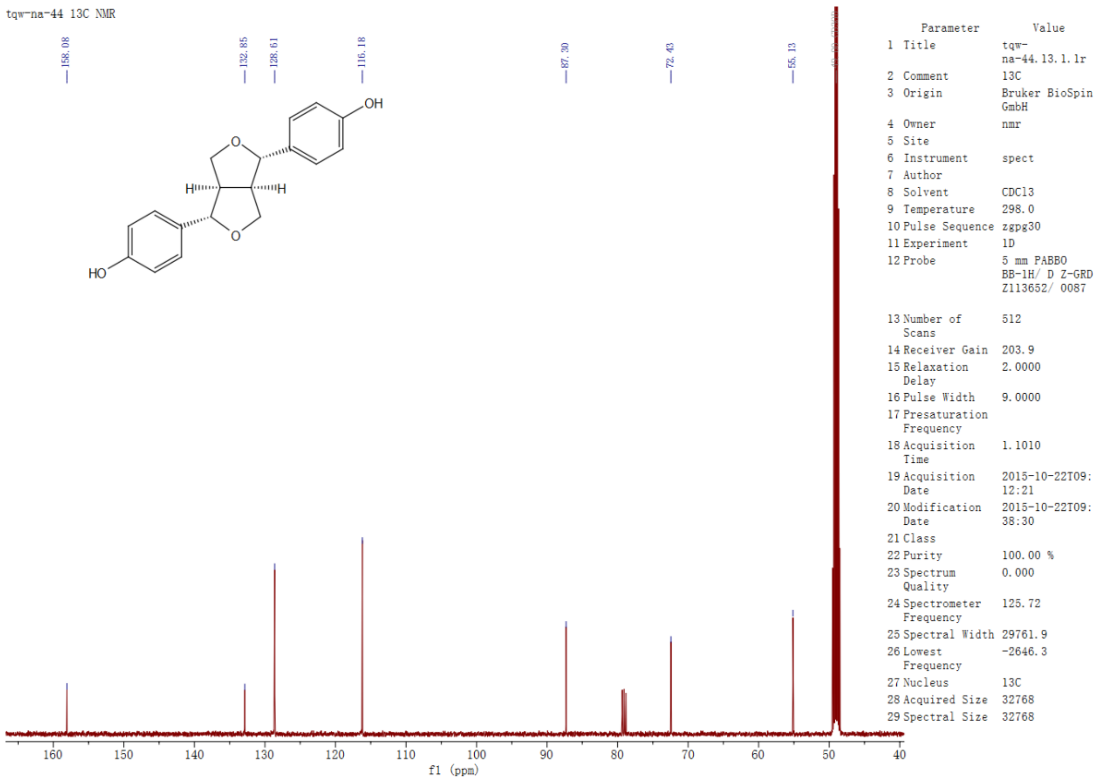

S24. ^{13}C NMR spectra of (+)-(1*R*,2*S*,5*R*,6*S*)-2,6-di(4'-hydroxyphenyl)-3,7-dioxabicyclo[3.3.0]octane (**5**) (125 MHz, Chloroform-*d*).

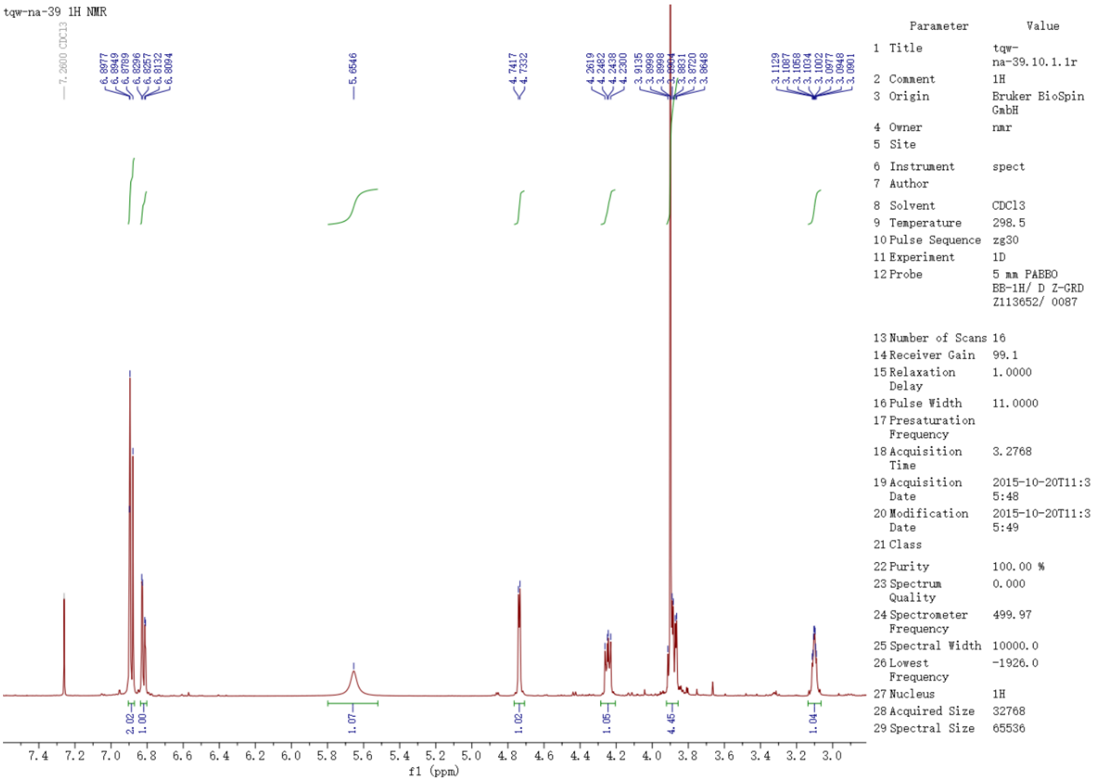

S25. ^{1}H-NMR spectra of (+)-pinoresinol (**6**) (500 MHz, Chloroform-*d*).

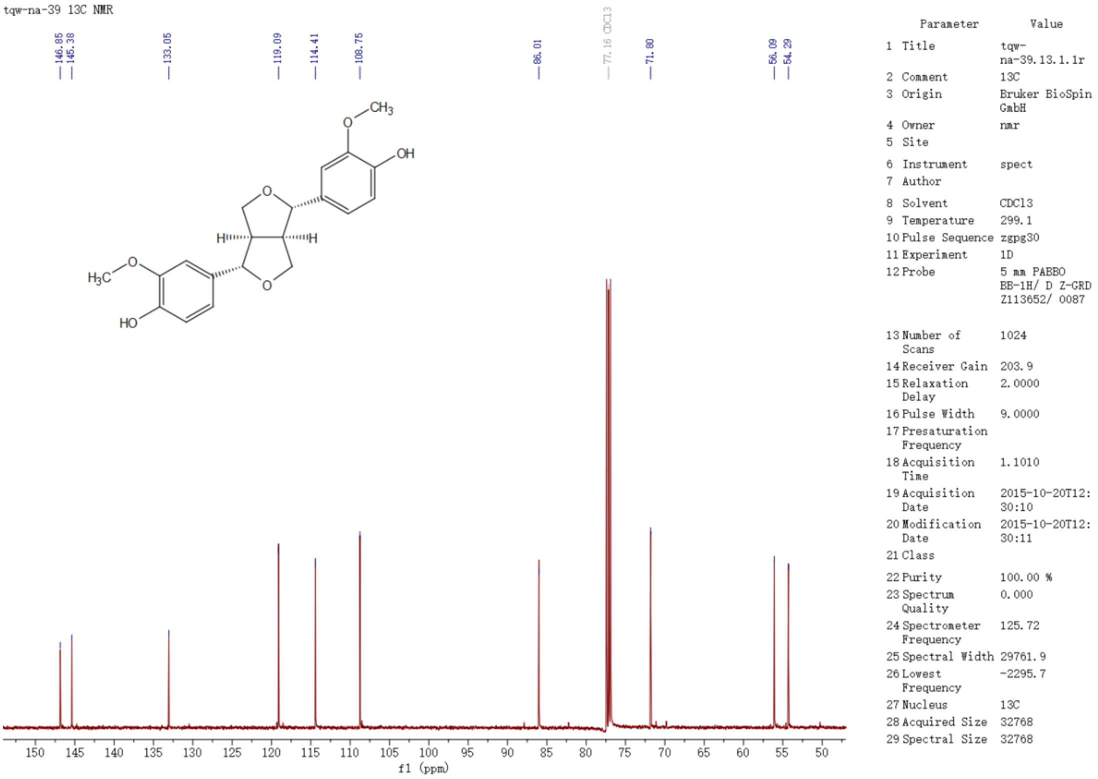

S26. ^{13}C NMR spectra of (+)-pinoresinol (**6**) (125 MHz, Chloroform-*d*).

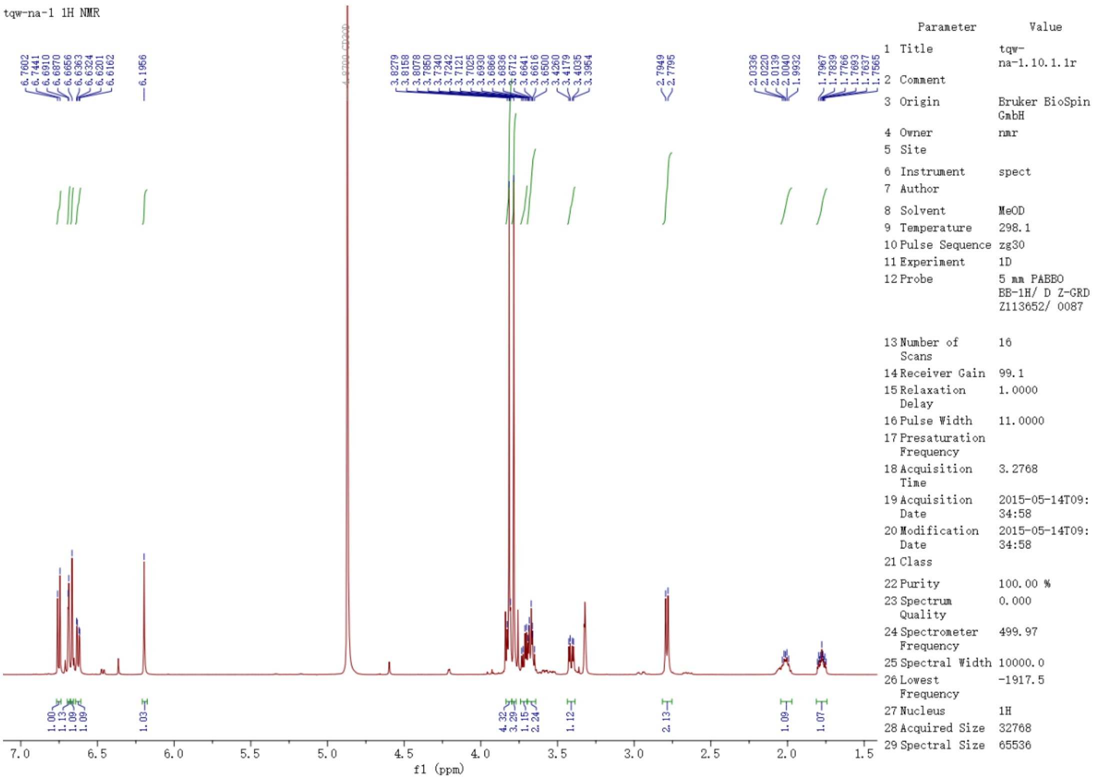

S27. ^{1}H-NMR spectra of (+)-isolariciresinol (**7**) (500 MHz, Methanol-d_4).

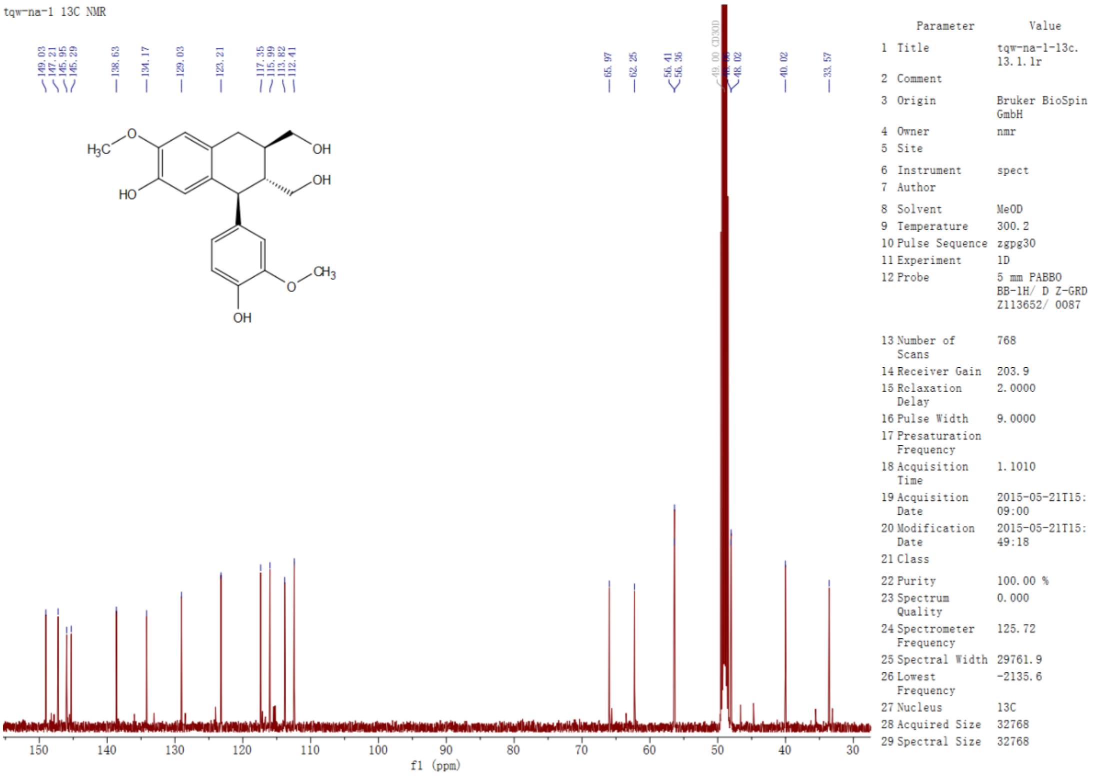

S28. ^{13}C NMR spectra of (+)-isolariciresinol (**7**) (125 MHz, Methanol-d_4).

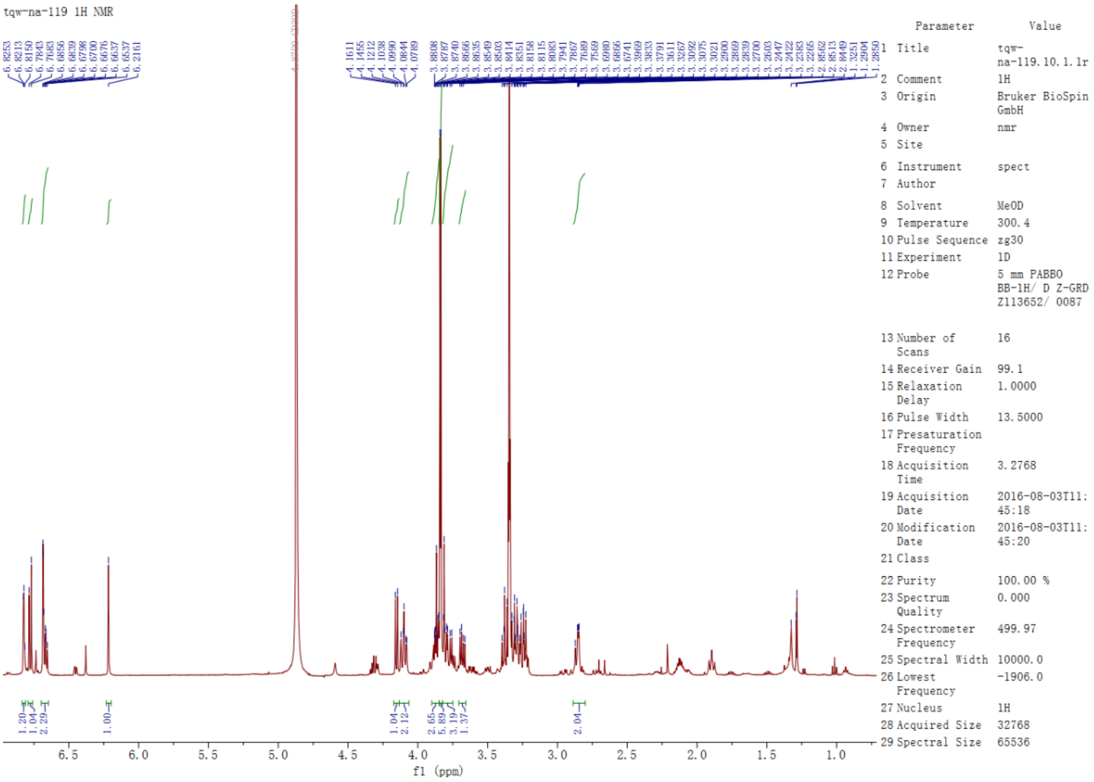

S29. ^{1}H-NMR spectra of (+)-isolariciresinol 3α-*O*-β-glucopyranoside (**8**) (500 MHz, Methanol-d_4).

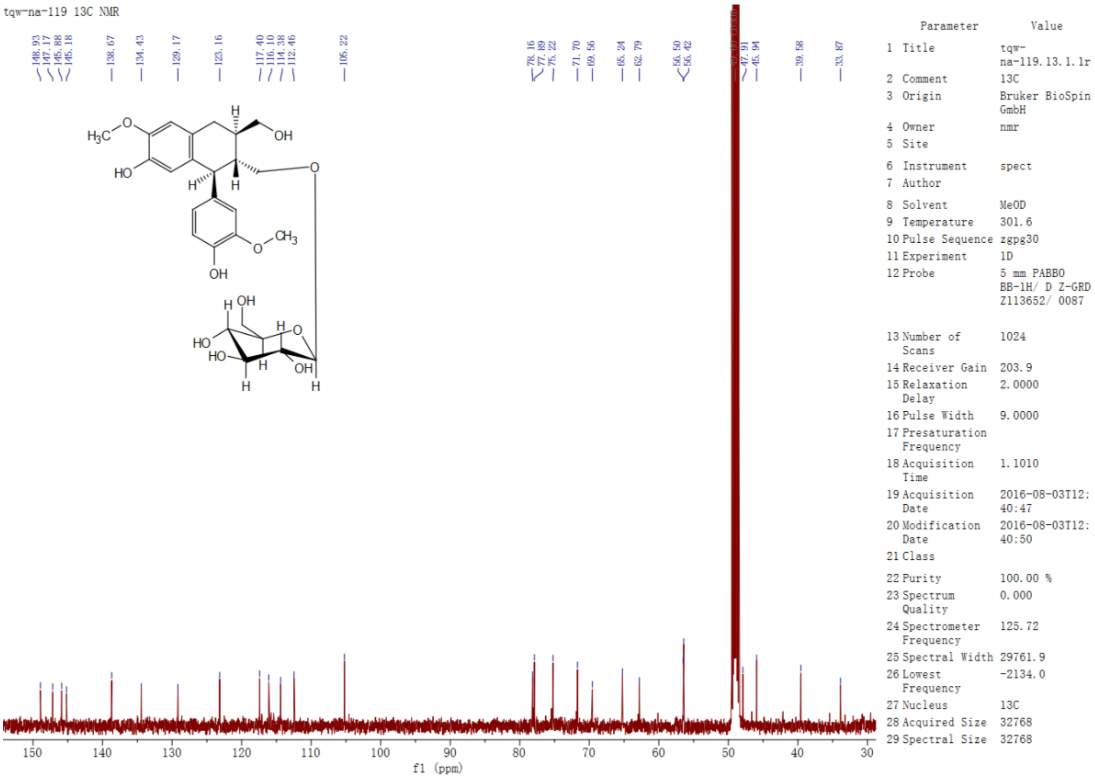

S30. ^{13}C NMR spectra of (+)-isolariciresinol 3α-*O*-β-glucopyranoside (**8**) (125 MHz, Methanol-d_4).

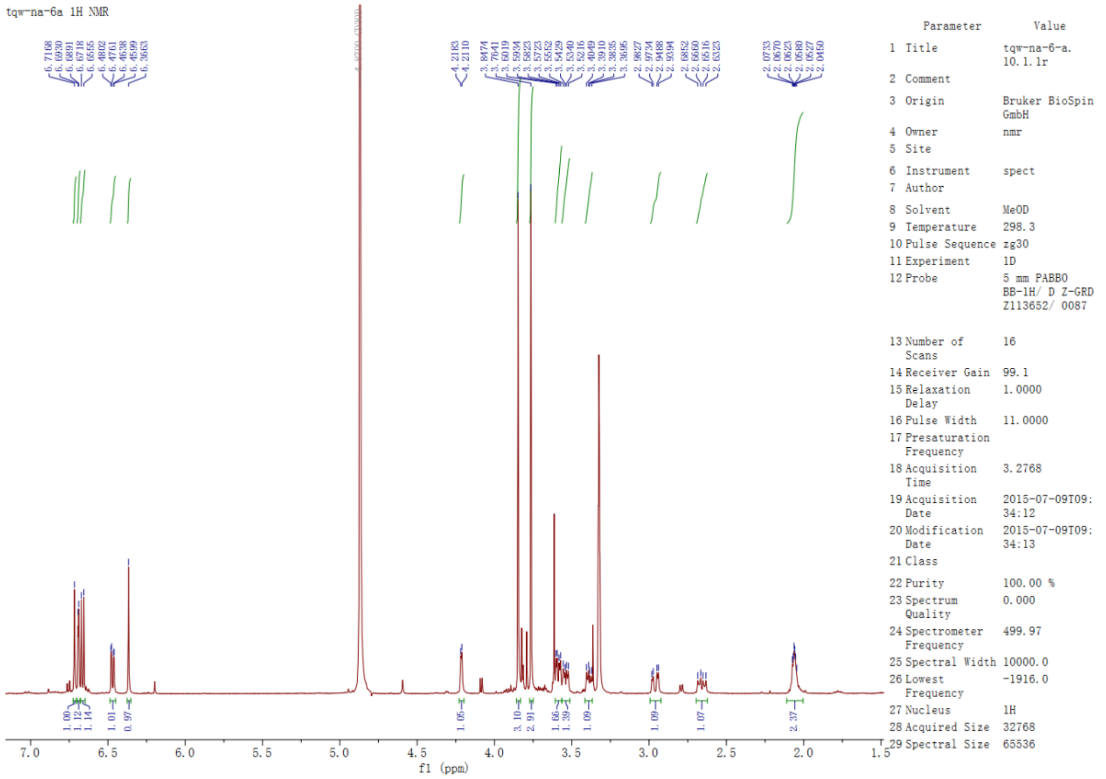

S31. ^{1}H-NMR spectra of burselignan (**9**) (500 MHz, Methanol-d_4).

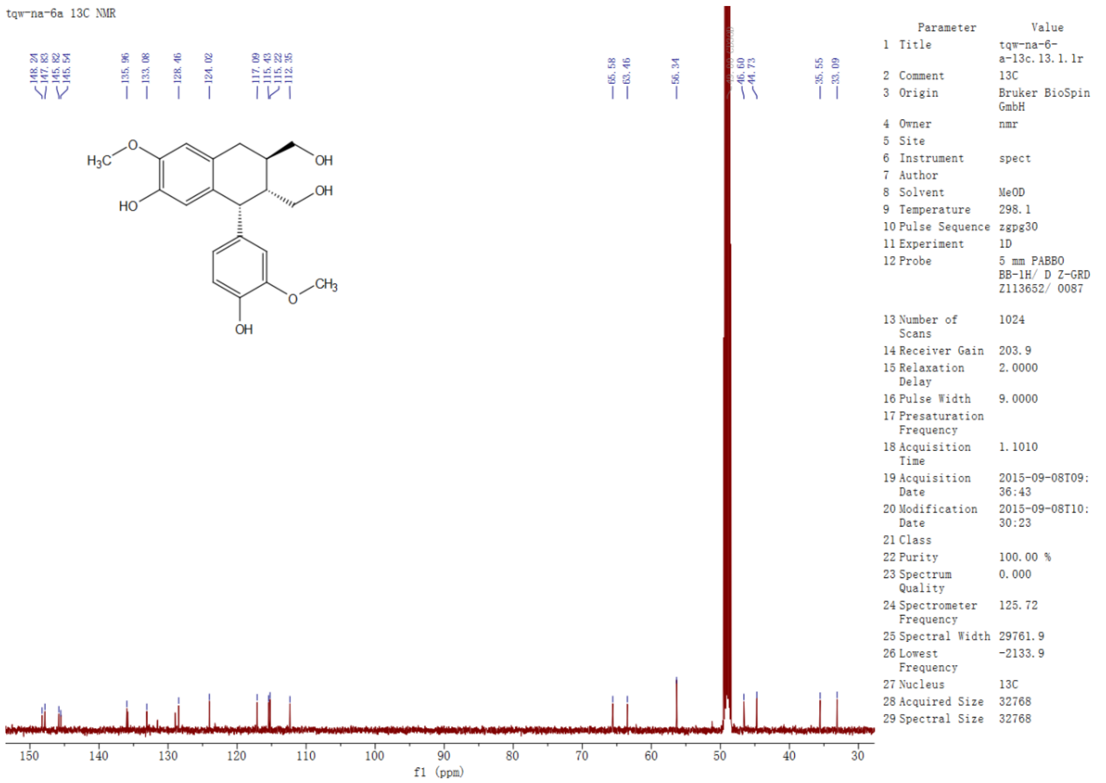

S32. ^{13}C NMR spectra of burselignan (**9**) (125 MHz, Methanol-d_4).

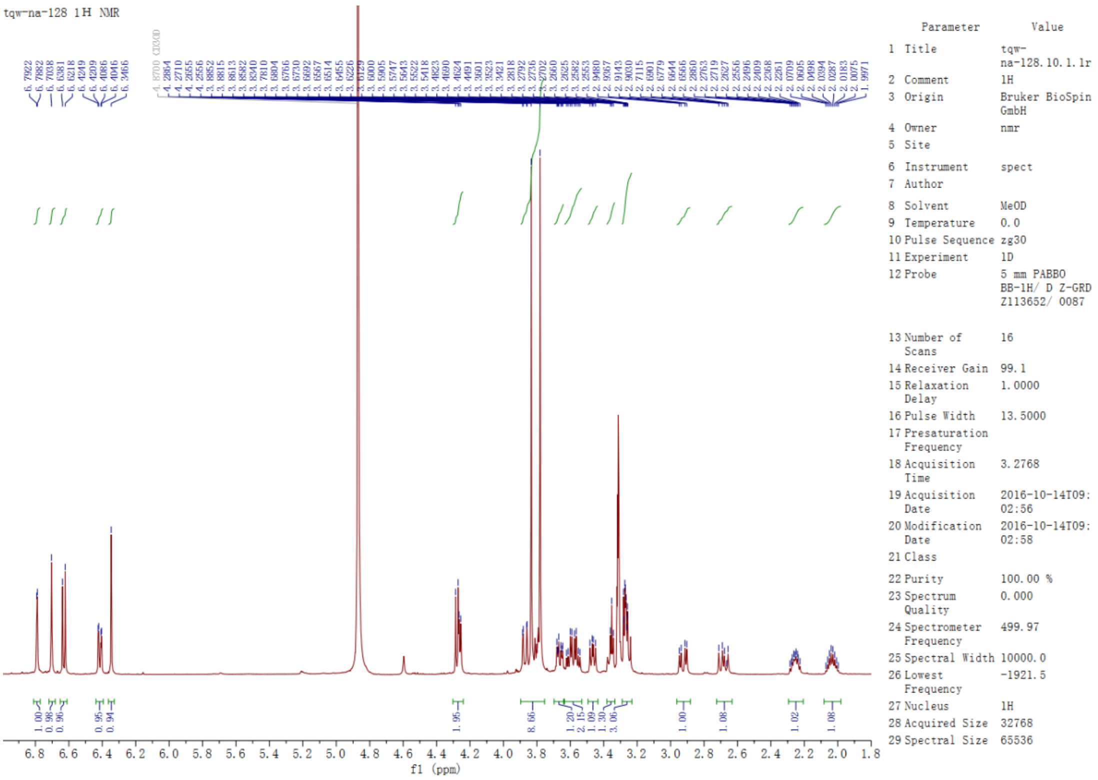

S33. ^{1}H-NMR spectra of densispicoside (**10**) (500 MHz, Methanol-d_4).

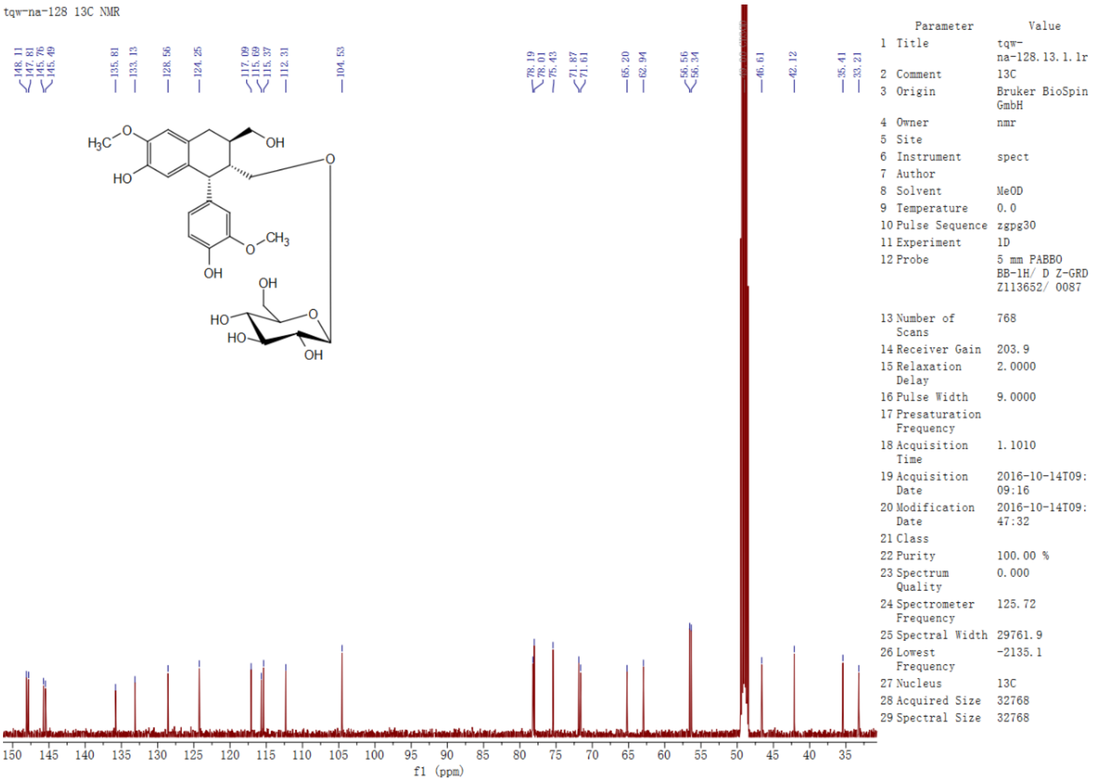

S34. ^{13}C NMR spectra of densispicoside (**10**) (125 MHz, Methanol-d_4).

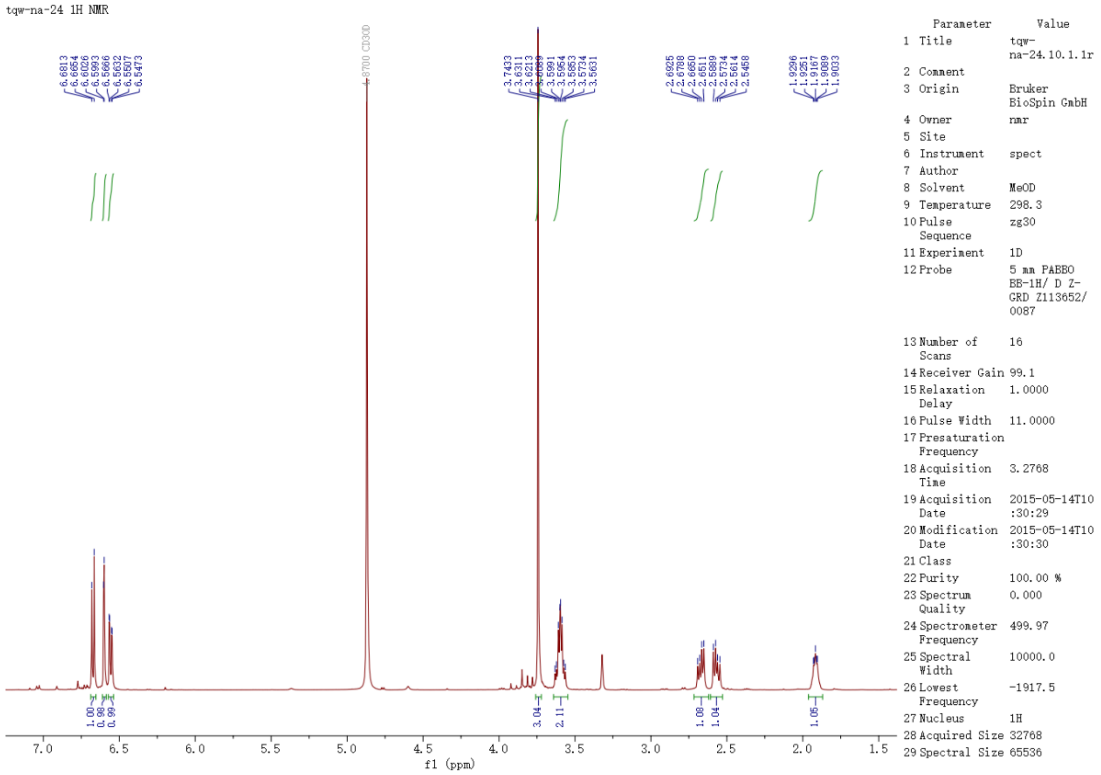

S35. ^{1}H-NMR spectra of secoisolariciresinol (**11**) (500 MHz, Methanol-*d*$_4$).

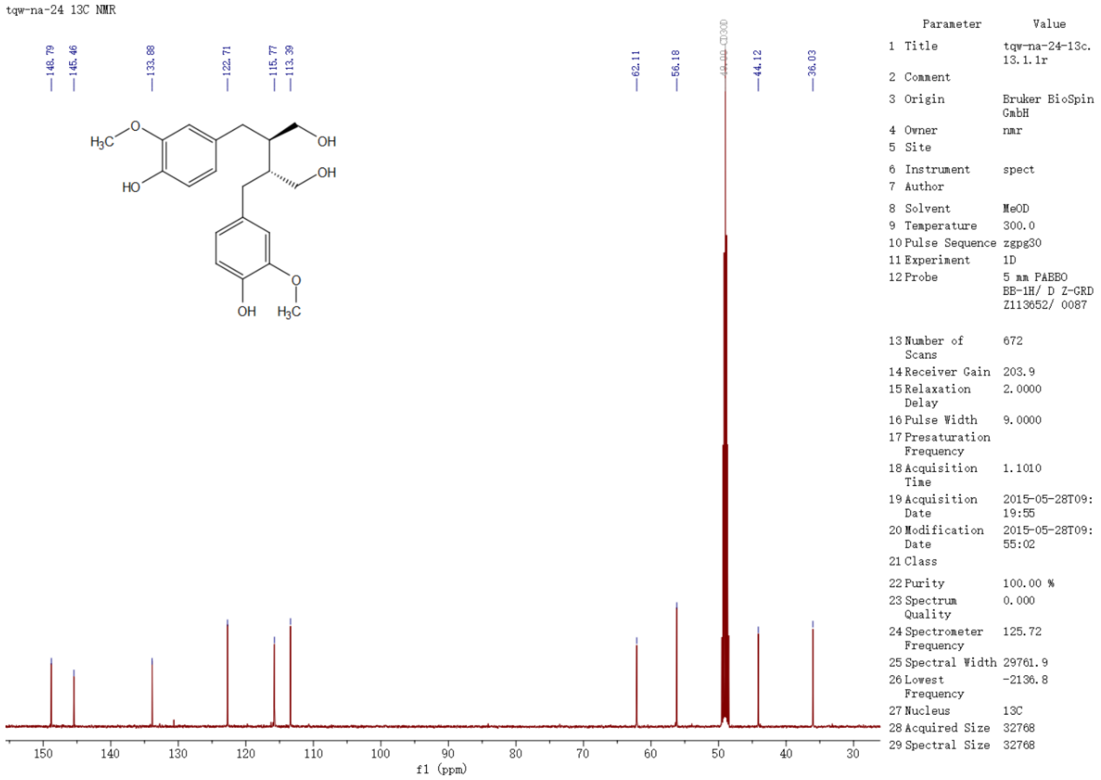

S36. ^{13}C NMR spectra of secoisolariciresinol (**11**) (125 MHz, Methanol-*d*$_4$).

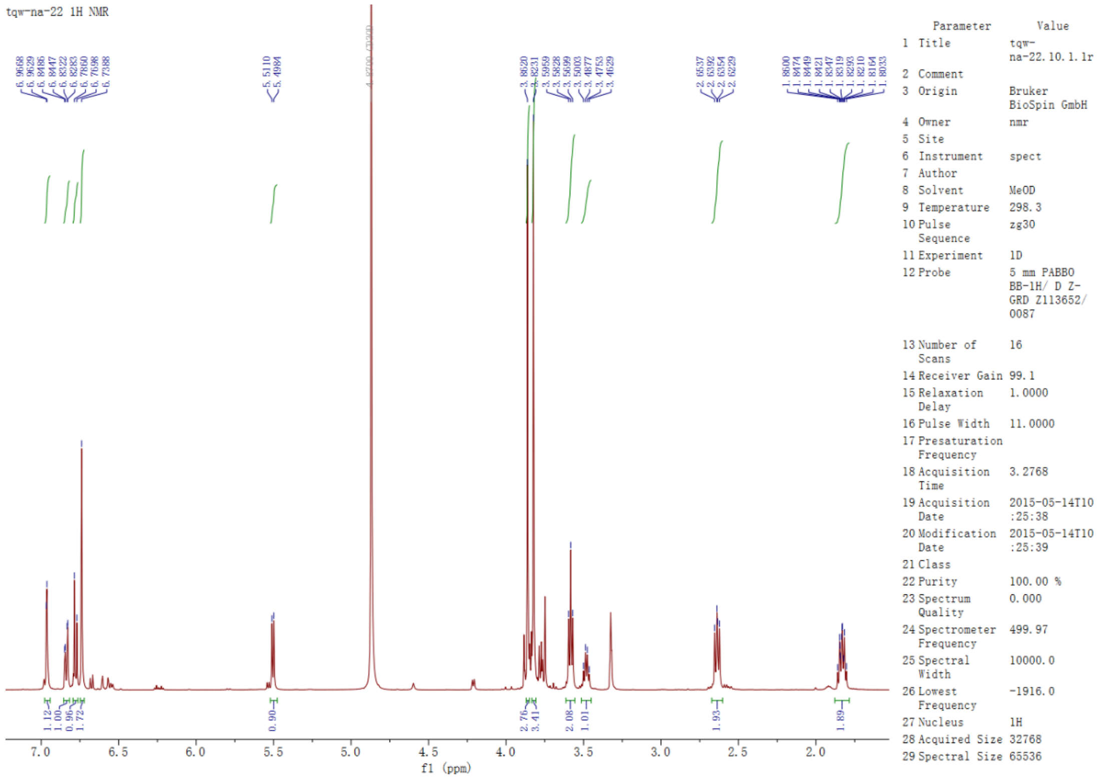

S37. ^{1}H-NMR spectra of dehydroconiferyl alcohol (**12**) (500 MHz, Methanol-d_4).

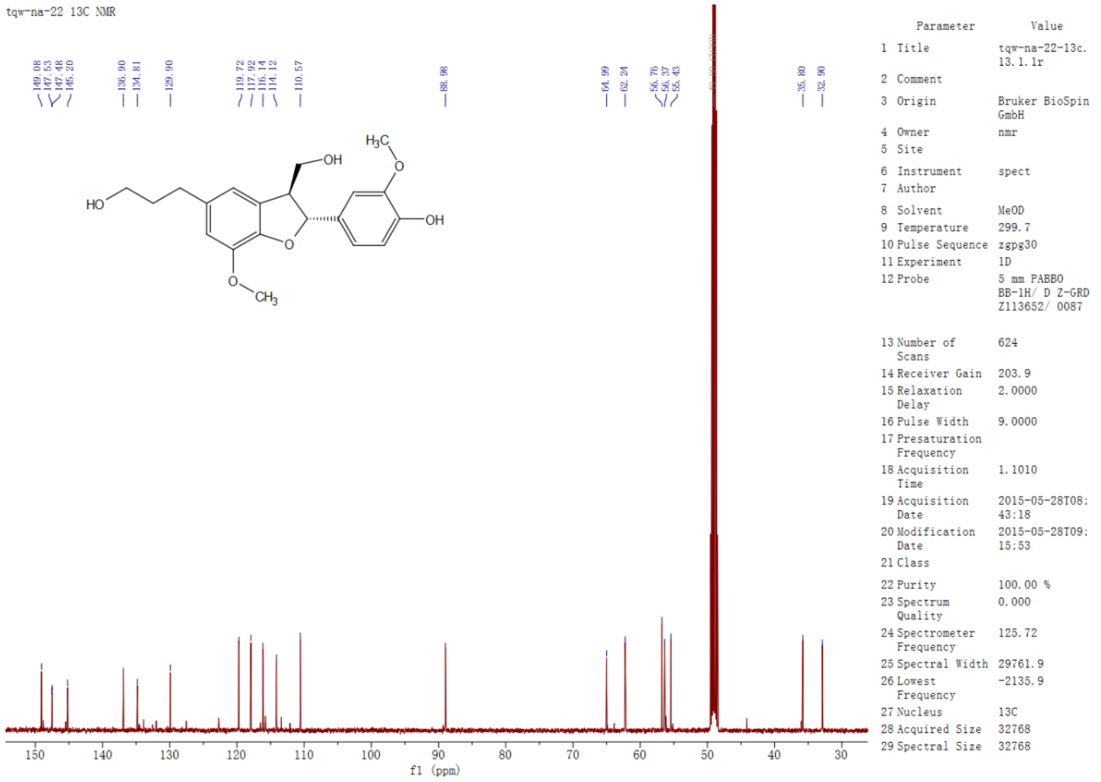

S38. ^{13}C NMR spectra of dehydroconiferyl alcohol (**12**) (125 MHz, Methanol-d_4).

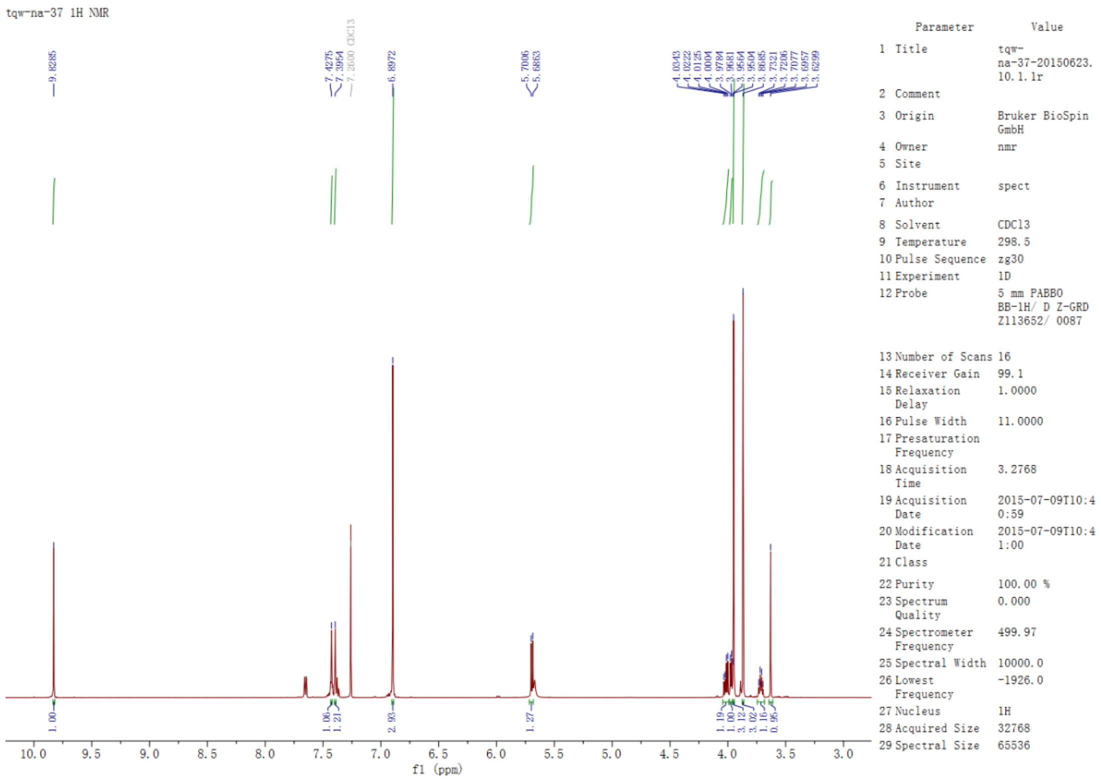

S39. ^{1}H-NMR spectra of curcasinlignan B (**13**) (500 MHz, Chloroform-*d*).

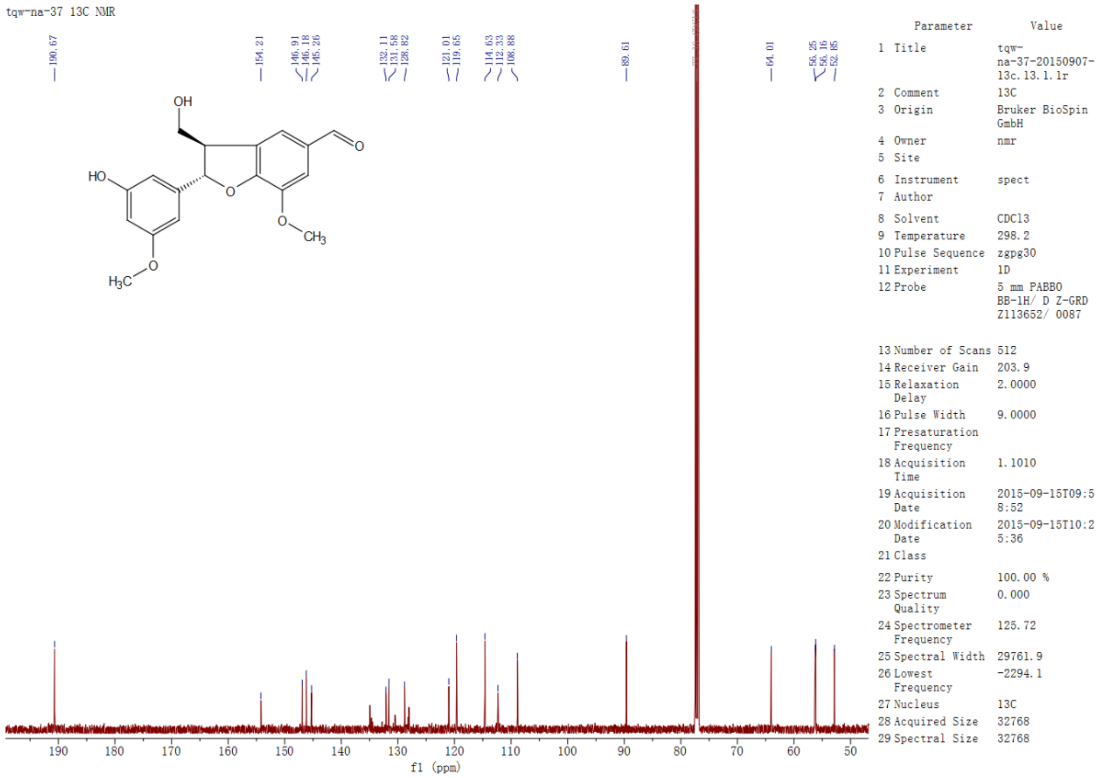

S40. ^{13}C NMR spectra of curcasinlignan B (**13**) (125 MHz, Chloroform-*d*).

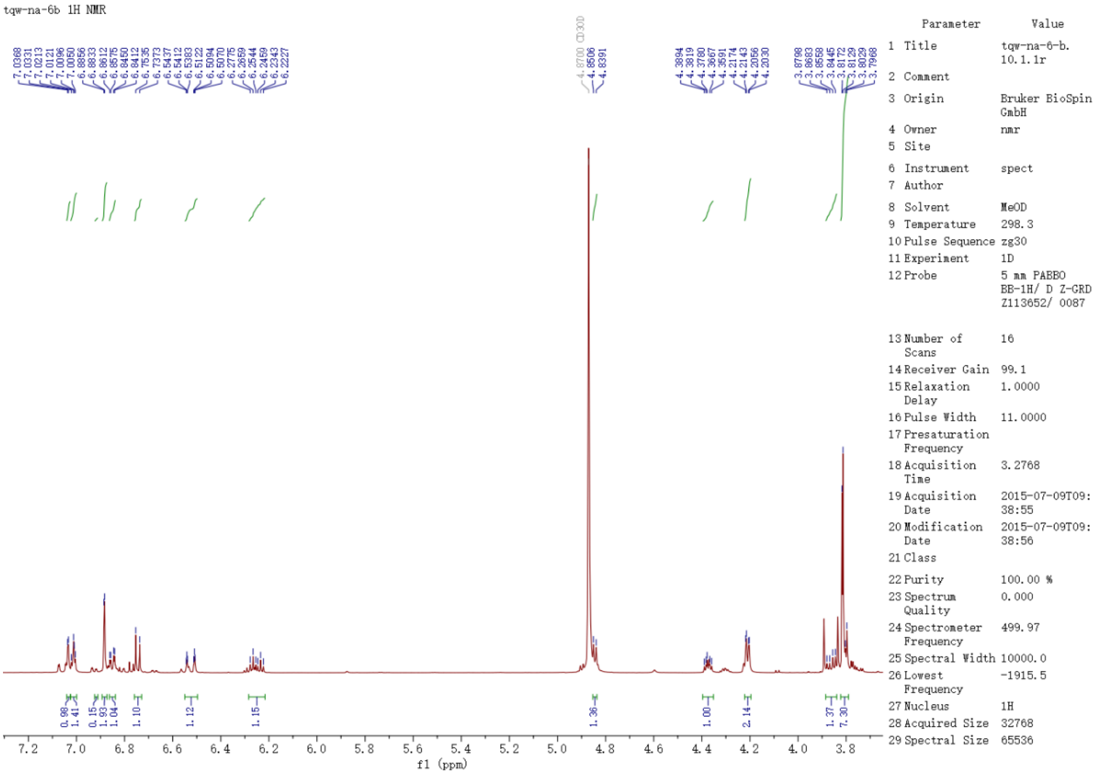

S41. ^{1}H-NMR spectra of *erythro*-guaiacylglycerol-*β*-*O*-4′-coniferyl ether (**14**) (500 MHz, Methanol-d_4).

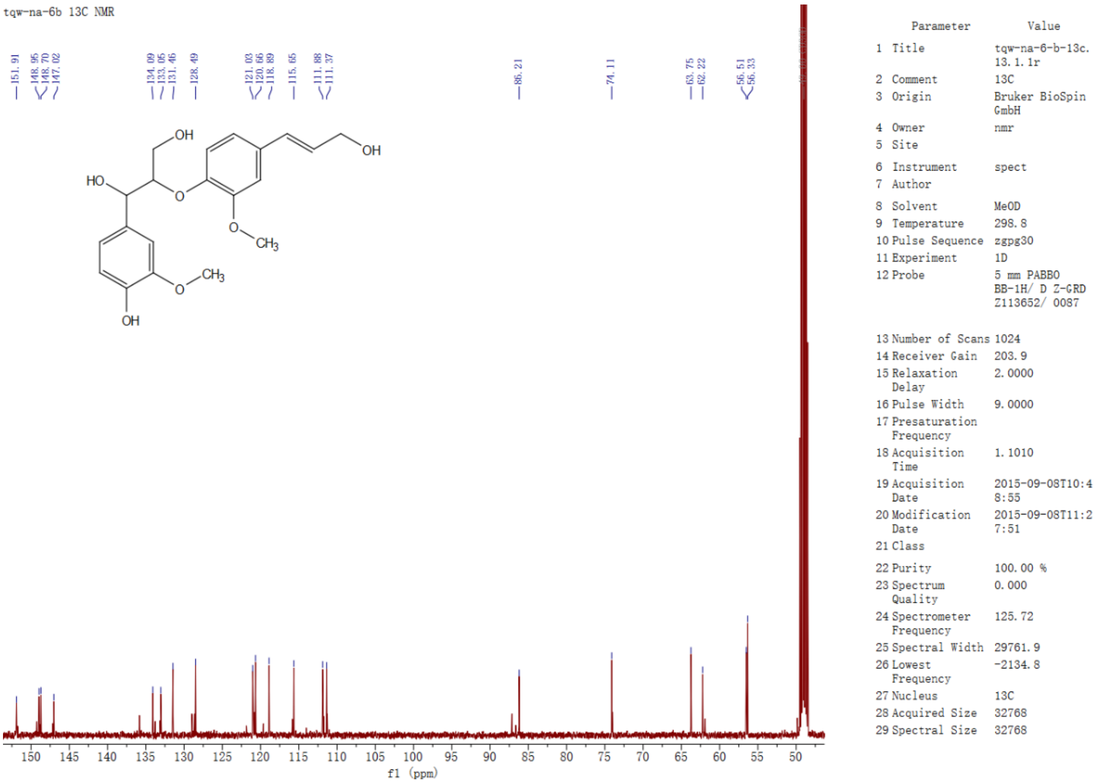

S42. ^{13}C NMR spectra of *erythro*-guaiacylglycerol-*β*-*O*-4′-coniferyl ether (**14**) (125 MHz, Methanol-d_4).

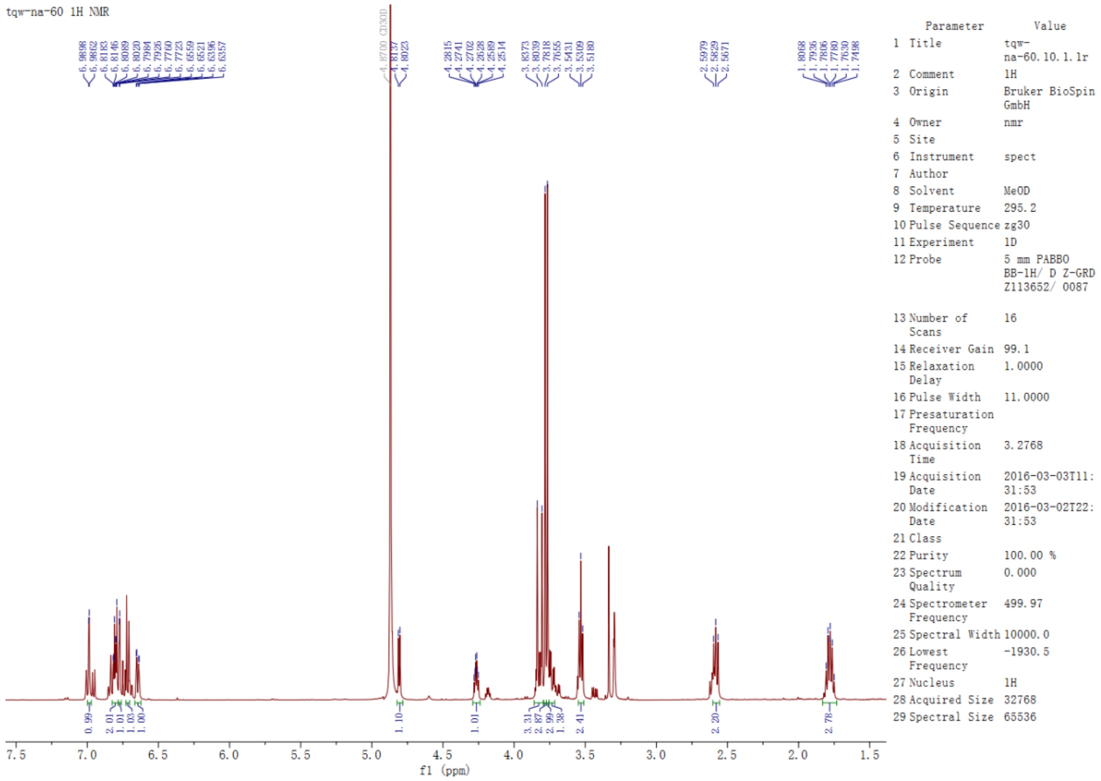

S43. ^{1}H-NMR spectra of 7*R*,8*R*-*threo*-4,7,9,9'-tetrahydroxy-3,3'-dimethoxy-8-*O*-4'-neolignan (**15**) (500 MHz, Methanol-d_4).

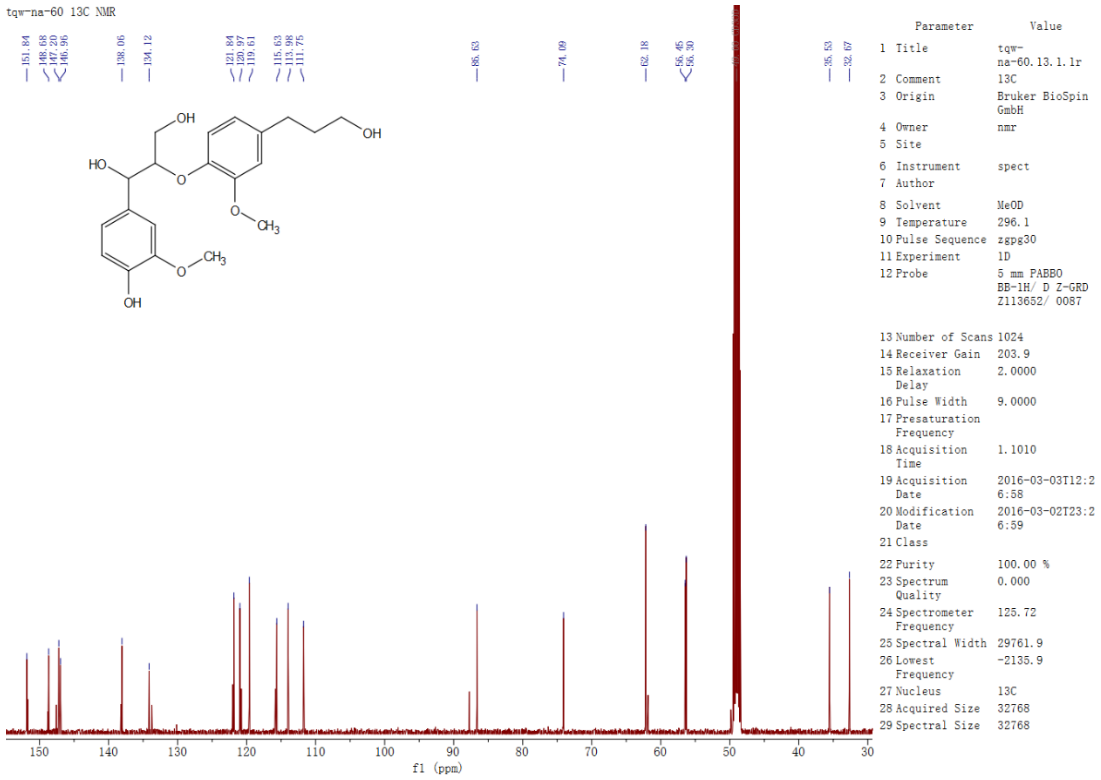

S44. ^{13}C NMR spectra of 7*R*,8*R*-*threo*-4,7,9,9'-tetrahydroxy-3,3'-dimethoxy-8-*O*-4'-neolignan (**15**) (125 MHz, Methanol-d_4).

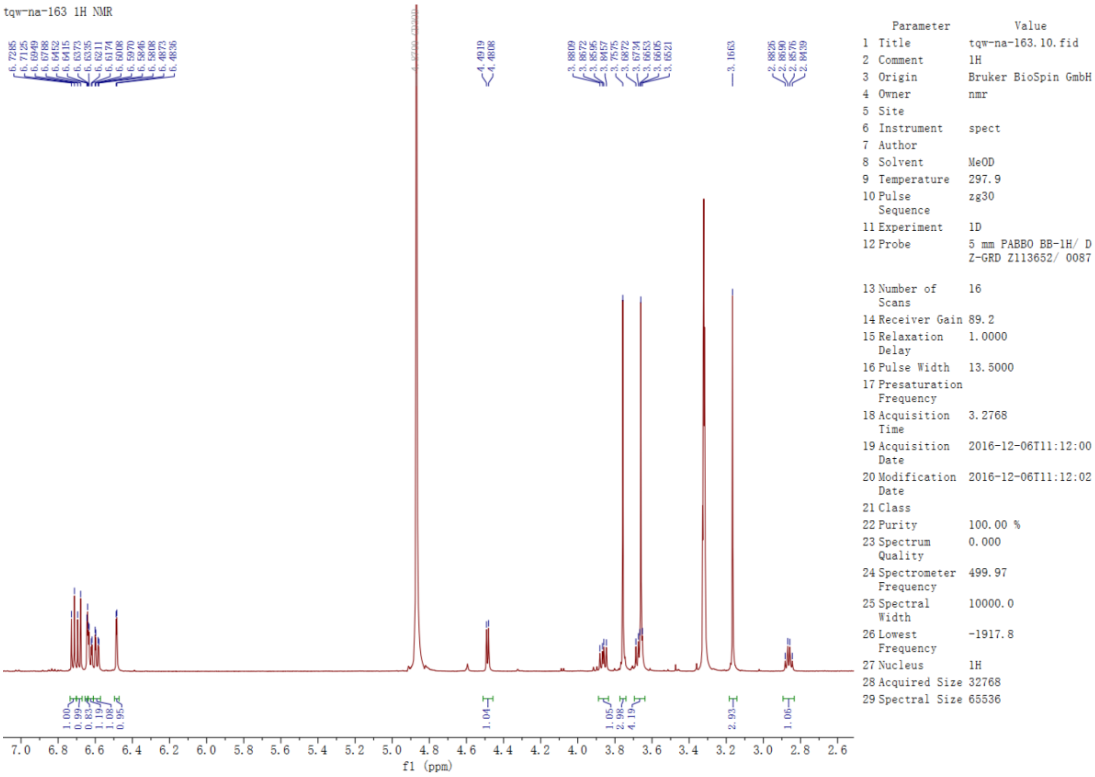

S45. ^{1}H-NMR spectra of *threo*-2,3-*bis*-(4-hydroxy-3-methoxyphenyl)-3-methoxypropanol (**16**) (500 MHz, Methanol-d_4).

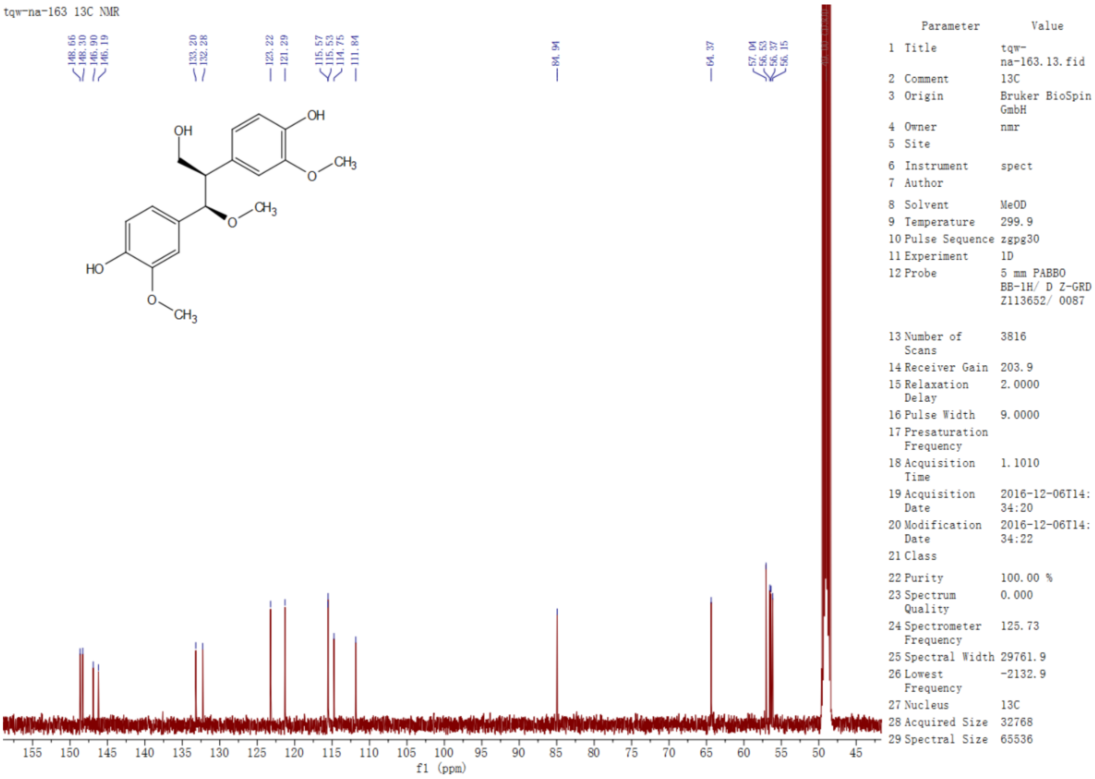

S46. ^{13}C NMR spectra of *threo*-2,3-*bis*-(4-hydroxy-3-methoxyphenyl)-3-methoxypropanol (**16**) (125 MHz, Methanol-d_4).
